# Estrogenic activity of E2-conjugated GLP-1 is mediated by intracellular endolysosomal acidification and estrone metabolism

**DOI:** 10.1016/j.molmet.2025.102136

**Published:** 2025-04-07

**Authors:** Callum Coupland, Na Sun, Ahmed Khalil, Özüm Ezgi Karaoglu, Arkadiusz Liskiewicz, Daniela Liskiewicz, Gerald Grandl, Seun Akindehin, Gandhari Maity, Bin Yang, Brian Finan, Patrick Knerr, Jonathan D. Douros, Axel Walch, Richard DiMarchi, Matthias H. Tschöp, Timo D. Müller, Aaron Novikoff

**Affiliations:** 1 Institute for Diabetes and Obesity, Helmholtz Munich, Neuherberg, Germany; 2 German Center for Diabetes Research (DZD), Neuherberg, Germany; 3 Analytical Pathology Unit, Helmholtz Munich, Neuherberg, Germany; 4 Department of Pharmacology, Experimental Therapy & Toxicology, Institute of Experimental & Clinical Pharmacology & Pharmacogenomics, Eberhard-Karls University Tübingen, Tübingen, Germany; 5 Department of Physiology, Faculty of Medical Sciences in Katowice, Medical University of Silesia, Katowice, Poland; 6 Institute of Physiotherapy and Health Sciences, Academy of Physical Education, Katowice, Poland; 7 Novo Nordisk Research Center Indianapolis, Indianapolis, IN, USA; 8 Dexatide LLC, Plainfield, IN, USA; 9 Eli Lilly and Company, Indianapolis, USA; 10 Indiana Biosciences Research Institute, Indianapolis, IN, USA; 11 Department of Chemistry, Indiana University, Bloomington, IN, USA; 12 Technische Universität, München, Germany; 13 Division of Metabolic Diseases, Department of Medicine, Technical University Munich, Munich, Helmholtz Munich, Neuherberg, Germany; 14 Walther-Straub-Institute for Pharmacology and Toxicology, Ludwig-Maximilians-University Munich, Germany

**Keywords:** Glucagon-like peptide 1, Estradiol, Peptide conjugation, Pharmacology, Metabolomics, Bioluminescence resonance energy transfer (BRET)

## Abstract

**Objective:**

Recent modifications to glucagon-like peptide 1 (GLP-1), known for its insulinotropic and satiety-inducing effects, have focused on conjugating small molecules to enable selective delivery into GLP-1R+ tissues to achieve targeted synergy and improved metabolic outcomes. Despite continued advancements in GLP-1/small molecule conjugate strategies, the intracellular mechanisms facilitating concurrent GLP-1R signaling and small molecule cargo release remain poorly understood.

**Methods:**

We evaluate an estradiol (E2)-conjugated GLP-1 (GLP-1-CEX/E2) for relative differences in GLP-1R signaling and trafficking, and elucidate endolysosomal dynamics that lead to estrogenic activity using various live-cell, reporter, imaging, and mass-spectrometry techniques.

**Results:**

We find GLP-1-CEX/E2 does not differentially activate or traffic the GLP-1R relative to its unconjugated GLP-1 backbone (GLP-1-CEX), but uniquely internalizes the E2 moiety and stimulates estrogenic signaling. Endolysosomal pH-dependent proteolytic activity likely mediates E2 moiety liberation, as evidenced by clear amplification in estrogenic activity following co-administration with lysosomal VATPase activator EN6. The hypothesized liberated metabolite from GLP-1-CEX/E2, E2-3-ether, exhibits partial estrogenic efficacy through ERα, and is predisposed toward estrone-3-sulfate conversion. Finally, we identify relative increases in intracellular E2, estrone, and estrone-3-sulfate following GLP-1-CEX/E2 incubation in GLP-1R+ cells, demonstrating proof-of-principle for desired cargo release.

**Conclusion:**

Together, our data suggest that GLP-1-CEX/E2 depends on GLP-1R trafficking and lysosome acidification for estrogenic efficacy, with a likely conversion of the liberated E2-3-ether metabolite into estrone-3-sulfate, resulting in a residual downstream flux into active estradiol. Our current findings aim to improve the understanding of small molecule targeting and the efficacy behind GLP-1/small molecule conjugates.

## Highlights

• E2 is conjugated to the C-terminal end of a CEX-modified GLP-1 (GLP-1-CEX/E2).
• GLP-1-CEX/E2 modifications minimally influence GLP-1R signalling and trafficking.
• GLP-1-CEX/E2 induces estrogenic activity not seen with GLP-1/CEX or native GLP-1.
• Endolysosomal acidification status correlates with GLP-1-CEX/E2 estrogenic activity.
• Intracellular increases of estradiol, estrone-3-suflate, and estrone with GLP-1-CEX/E2.

## Graphical abstract

**Figure:**
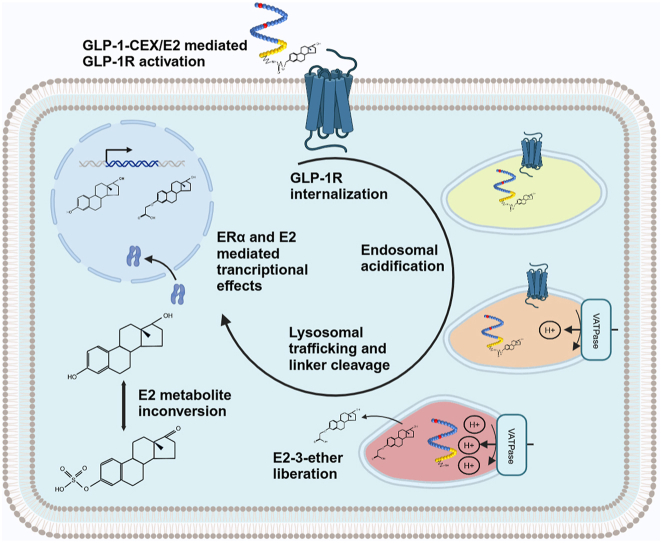

## 1 Introduction

Anti-obesity therapies have significant potential to mitigate metabolic syndrome and protect against cardiometabolic diseases. Pharmacological therapies are increasingly favored over invasive options like bariatric surgery due to case-specific scalability, cost-effectiveness, reversibility, and absence of post-surgical complications. One promising pharmacological approach involves the use of glucagon-like peptide 1 (GLP-1), an endogenous peptide known for its insulinotropic and satiety-inducing effects [[1], [2], [3]]. Recent synthetic optimizations, which include fatty acylation and amino acid substitutions, have significantly improved the stability and circulating half-life of GLP-1, thereby enhancing its therapeutic effectiveness [1,2,4].

Improvements in the circulating stability of GLP-1 have enabled a deeper understanding of the target tissues and mechanisms mediating its insulinotropic and satiety-inducing effects [5,6]. The overlap of GLP-1 receptor (GLP-1R) expression with that of other therapeutic disease-related targets has spurred the development of covalent conjugation of nuclear hormones or small molecules onto the C-terminal end of the GLP-1 peptide [[7], [8], [9], [10], [11], [12]]. These conjugates aim to selectively deliver small molecular cargo into GLP-1R expressing tissues, synergistically engaging parallel GLP-1R and small molecule activities, and improving the therapeutic window of the small molecule by avoiding off-target effects in tissues lacking the GLP-1 receptor [13,14].

Inspired by antibody-drug conjugates, linker technologies binding peptide carriers to small molecules are designed for optimal stability in circulation while retaining cleavability within target cells, ensuring tissue-specific efficacy [13]. A variety of conjugation chemistries offer alternative chemical, enzymatic, and plasma stabilities, while also containing differential modes-of-action and predisposition toward intracellular cleavability [13].

Several innovative GLP-1/small molecule conjugates have been developed, each offering distinct therapeutic advantages. GLP-1/estradiol was the first proof-of-concept for GLP-1-mediated small molecule delivery, enabling targeted estradiol uptake into GLP-1R^+^ tissues while mitigating off-target, dose-dependent oncogenic and uterine hypertrophic risks - ultimately leveraging the beneficial overlapping insulinotropic and satiety actions of GLP-1 and estradiol in pancreatic β-cells and hypothalamic feeding centers [7,12,15,16]. GLP-1 conjugation to the potent glucocorticoid dexamethasone (GLP-1/dexamethasone) was designed for and achieved targeted anti-inflammatory effect within GLP-1R^+^ hypothalamic central nervous system (CNS) tissue [10,17]. Similarly, GLP-1/tesaglitazar, which links GLP-1 to the insulin sensitizing α/γ peroxisome proliferator-activated receptor (PPAR) dual-agonist tesaglitazar, exhibited strong anti-diabetic efficacy while potentially repurposing the small molecule's safety profile, addressing potential renal concerns that contributed to its discontinuation in phase II/III clinical trials [9]. More recently, GLP-1/MK-801 has highlighted the potential for targeted N-methyl-d-aspartate (NMDA) receptor antagonism in GLP-1R^+^ feeding centers to counteract compensatory neural adaptations that drive increased food-seeking behavior, while avoiding the adverse behavioral and physiological effects attributed to loose systemic MK-801 treatment [11]. Despite growing interest in GLP-1/small molecule conjugates, the intracellular spatiotemporal signaling and trafficking dynamics of these agonists at the GLP-1R, as well as the intracellular events governing linker cleavage, small molecule liberation, and ultimately, the small molecule metabolites that result from these processes, remain incompletely understood.

Small molecule conjugation to various G-protein coupled receptor (GPCR) peptide agonists will continue evolving, improving in both strategy and therapeutic effectiveness. Within currently available conjugates, the targeted delivery of estradiol (E2) into GLP-1R expressing tissues, such as GLP-1R^+^ glucoregulatory pancreatic β-cells and satiety-inducing neuronal populations, has been shown to enhance pancreatic β-cell pro-survivability and synergistically reduce food intake, body weight, and blood glucose compared to structurally-matched GLP-1 controls [7,12,15,16,18]. To explore how these synergies occur, we sought to better understand the underlying intracellular GLP-1R and E2 pharmacodynamics elicited by the E2-conjugated GLP-1 (GLP-1-CEX/E2) relative to the matched C-terminally extended GLP-1 backbone (GLP-1-CEX) and native GLP-1 (7-36)amide. The GLP-1-CEX backbone includes several modifications: a 2-aminoisobutyric acid (Aib) substitution at position 2 for *in vivo* protection against dipeptidyl peptidase-IV inactivation; a nine amino acid C-terminal extension derived from the GLP-1 paralogue exendin-4; addition of a C-terminal lysine amide to connect the GLP-1 peptide and linker/E2 moiety; and a glutamic acid substitution at position 16 to retain maximal GLP-1 potency [7]. The linker connecting the GLP-1-CEX C-terminal lysine to estradiol consists of a short carbon chain and an amide bond at the 3-hydroxyl group of E2, forming an amide/ether linker. While not the only meta-stable linker available for conjugation, the amide-based linker offers potential proteolytic susceptibility to endolysosomal peptidases that can act to liberate a resultant small molecule [13]. Hydrolytic cleavage of this particular amide/ether linker in GLP-1-CEX/E2 likely results not in native estradiol but in a derivative of E2, hypothesized to be estradiol 3-carboxymethyl ether (E2-3-ether) [7].

In this study we employed bioluminescence resonance energy transfer (BRET) assays, cellular imaging, nuclear hormone activity reporters, and spatial metabolomics to understand the step-by-step intracellular processes regulating the estrogenic efficacy of GLP-1-CEX/E2. Our findings reveal that GLP-1 (7-36)amide, GLP-1-CEX, and GLP-1-CEX/E2 do not greatly differ in GLP-1 receptor activation or internalization, and only differentiate by a mild E2 conjugation-specific decrease in lysosomal GLP-1R accumulation. This suggests GLP-1 C-terminal E2 conjugation minimally impacts the canonical dynamics of GLP-1R agonism relative to GLP-1-CEX. Further, we demonstrate the estrogenic activity of GLP-1-CEX/E2 to be GLP-1R-dependent and amplified by pharmacological increases in lysosomal acidification. This suggests pH-dependent intracellular proteolytic cleavage of the amide/ether linker likely mediates the subsequent amplitude of estradiol activity. Importantly, using matrix-assisted-laser-desorption-ionization imaging mass spectrometry (MALDI-IMS), we identify that GLP-1-CEX/E2 induces increases in E2 and the E2-intermediates estrone and estrone-3-sulfate.

## 2 Results

### 2.1 Covalent conjugation of 17β-estradiol to the C-terminal end of a GLP-1 analogue does not inhibit GLP-1R signaling or internalization

**Figure 1:**
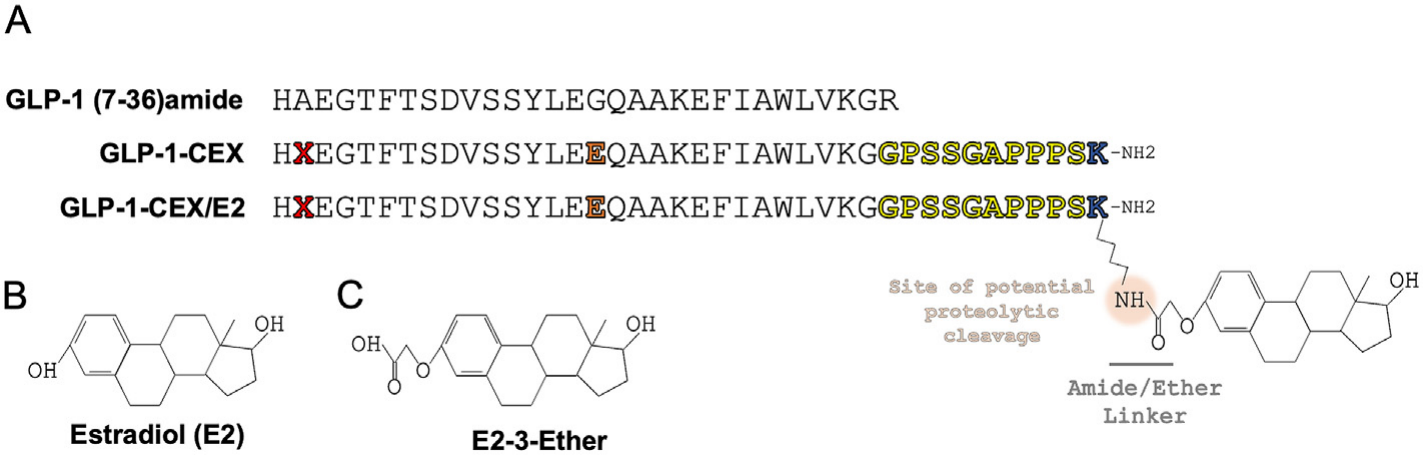
**(A)** Amino acid sequence of GLP-1R peptide agonists used, first showing endogenous active GLP-1 (7-36)amide, second the Aib2/Glu16/CEX modified GLP-1-CEX, and then the GLP-1-CEX/E2 peptide-nuclear hormone conjugate, containing 17β-estradiol covalently linked to the C-terminal end of GLP-1-CEX via an amide-ether linker. **(B)** 17β-estradiol, and **(C)** the projected liberated metabolite, E2-3-ether, following proteolytic hydrolysis of the amide/ether linker at the amide site.

**Figure 2:**
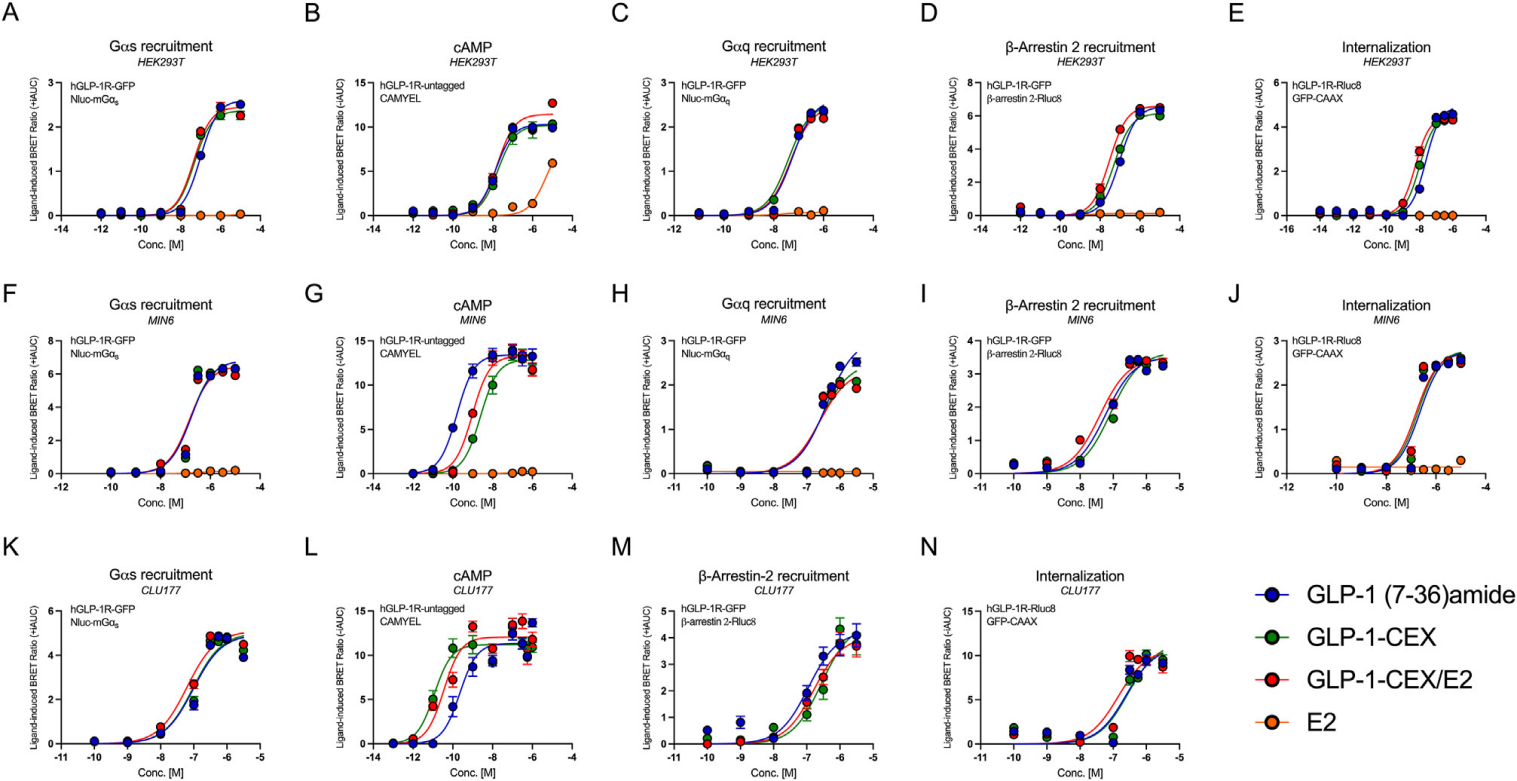
GLP-1R signaling and internalization dynamics recorded using BRET in response to varied dosages of GLP-1 peptides (GLP-1 (7-36)amide (blue), GLP-1-CEX (green), and GLP-1-CEX/E2 (red)), using a dose–response of E2 (orange) as the negative control. Figures A–E were carried out in transfected HEK293T cells; Figures F–J, in the mouse β-cell MIN6 cell model; and Figures K–N in the mouse hypothalamic neuron CLU177 cell model. **(A)** Ligand-induced Nluc-mGα_s_ recruitment to GLP-1R-GFP. **(B)** cAMP production using unimolecular CAMYEL BRET indicator. **(C)** Nluc-mGα_q_ recruitment to GLP-1R-GFP. **(D)** β-arrestin-2-Rluc8 recruitment to GLP-1R-GFP. **(E)** GLP-1R-Rluc8 internalization away from the stationary plasma membrane marker GFP-CAAX. **(F)** Nluc-mGα_s_ recruitment, **(G)** cAMP production, **(H)** Nluc-mGα_q_ recruitment, **(I)** β-arrestin-2-Rluc8 recruitment, **(J)** GLP-1R-Rluc8 internalization in the mouse β-cell MIN6 cell model. **(K)** Nluc-mGα_s_ recruitment, **(L)** cAMP production, (**M)** β-arrestin-2-Rluc8 recruitment, **(N)** GLP-1R-Rluc8 internalization in the mouse hypothalamic neuron CLU177 model. Values were derived from the iAUC of a temporal response for each agonist, dose–response values were obtained by using a three-parameter non-linear regression. Three independent experiments were performed with at least two technical replicates per group. (For interpretation of the references to color in this figure legend, the reader is referred to the Web version of this article).

**Supplementary Figure 1:**
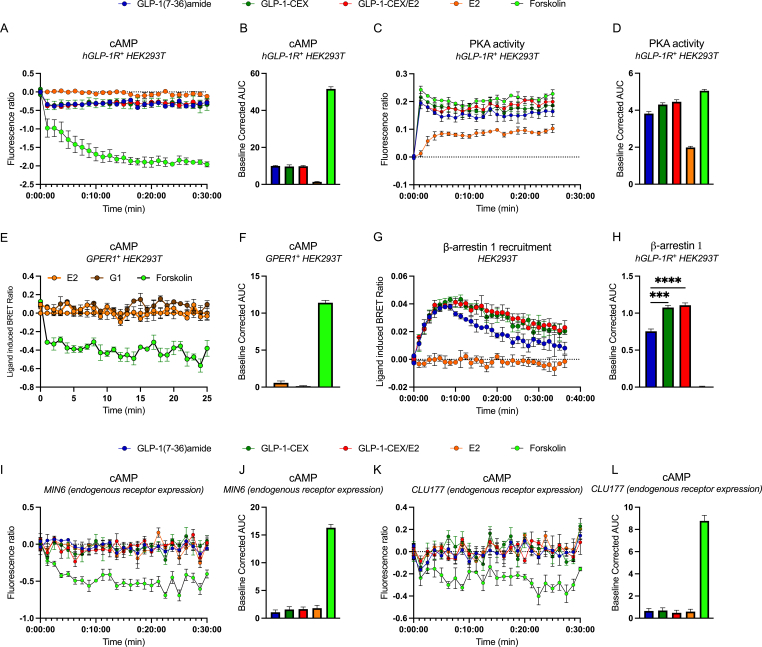
Temporal and AUC quantifications of **(A**–**B)** cAMP production and **(C**–**D)** PKA activity in response to 1 μM administration of GLP-1 (7-36)amide, GLP-1-CEX, GLP-1-CEX/E2, E2, and forskolin (50 μM) in hGLP-1R + HEK293T cells. **(E)** Temporal and **(F)** AUC quantifications of cAMP production in response to E2, G1 (GPER1 selective agonist), and forskolin (50 μM) in GPER1^+^ HEK293T cells. **(G)** Temporal and **(H)** AUC quantifications of β-arrestin 1 recruitment in response to GLP-1 (7-36)amide, GLP-1-CEX, GLP-1-CEX/E2, and E2 in hGLP-1R + HEK293T cells. Temporal and AUC quantifications of cAMP production in response to 1 μM administration of GLP-1 (7-36)amide, GLP-1-CEX, GLP-1-CEX/E2, E2, and forskolin (50 μM) in **(I**–**J)** MIN6 and **(K**–**L)** CLU177 cells lacking synthetic overexpression of GLP-1R. Data is vehicle subtracted. +iAUC graphs display mean ± SEM for each agonist. Experiment was carried out three times in HEK293T cells with at least two technical replicates per agonist. ∗p ≤ 0.05, ∗∗p ≤ 0.01, ∗∗∗p ≤ 0.001, ∗∗∗∗p ≤ 0.0001 using an unpaired two-tailed t test, or a one-way ANOVA with Tukey correction for multiple comparisons.

We examined multiple GLP-1R signaling and internalization modalities following exposure to the tested agonists GLP-1 (7-36)amide, GLP-1-CEX, and GLP-1-CEX/E2 (Figure 1A). Using live-cell bioluminescence resonance energy transfer (BRET), hGLP-1R^+^ HEK293T cells did not differentiate with respect to GLP-1R Gαs/Gαq recruitment or cAMP production in both Emax and potency between agonists (Figure 2A–C, Table 1). Saturation of the cAMP sensor was not achieved as demonstrated by 50 μM forskolin treatment (Supplementary Figs. 1A–B) These findings of signaling-associated equivalence between GLP-1-based agonists were further verified by downstream effector protein kinase A (PKA) activity (Supplementary Figs. 1C–D). G-protein coupled estrogen receptor 1 (GPER1) is proposed to be a Gαs-coupled GPCR responsive to estrogen or its specific agonist G1; however we did not observe E2-or G1-driven cAMP production (Supplementary Figs. 1E–F). While this may seem counterintuitive, it aligns with recent literature [19] and suggests cAMP production driven by GLP-1-CEX/E2 is unlikely to be supplemented by GPER1. Minimal differences in potency and comparable maximal efficacies were observed for GLP-1R β-arrestin 2 (βarr2) recruitment (Figure 2D) and GLP-1R internalization (Figure 2E) across all GLP-1-based ligands. Further, CEX-containing GLP-1 analogs showed no significant differences in β-arrestin 1 recruitment at 1 μM but exhibited somewhat greater effects than GLP-1 (7-36)amide (Supplementary Figs. 1G–H). These findings suggest that in a generally accepted model for GPCR signaling and trafficking (hGLP-1R^+^ HEK293T), estradiol conjugation to the C-terminal end of GLP-1-CEX does not significantly alter intracellular GLP-1R pharmacodynamics. In mouse pancreatic β-cell MIN6 cells transiently transfected to express hGLP-1R, as endogenous receptor expression does not confer a quantifiable cAMP response, all GLP-1-based ligands exhibit equal potency and efficacy for Gαs, Gαq, and βarr2 recruitment, and GLP-1R internalization (Figure 2F, H-J; Supplementary Figs. 1I–J; Table 1). However, native GLP-1 (7-36)amide exhibits superior potency for cAMP production relative to both GLP-1-CEX and GLP-1-CEX/E2 (Figure 2G, Table 1), potentially suggesting an unknown influence in this model driven by either peptide structure or cell-specific system bias. In CLU177 cells, a mouse-specific hypothalamic neural cell line transiently transfected to express hGLP-1R to allow for a quantifiable cAMP response, GLP-1-CEX and GLP-1-CEX/E2 exhibit equal potency and efficacy in both Gαs and β-arr2 recruitment, and GLP-1R internalization relative to GLP-1 (7-36)amide (Figure 2K, M-N; Supplementary Figure K–L; Table 1). However, in hGLP-1R^+^ CLU177 cells, cAMP production potencies for GLP-1-CEX and GLP-1-CEX/E2 were superior to that of GLP-1 (7-36)amide (Figure 2L; Table 1), suggesting another potential unknown influence seemingly opposite to that seen in MIN6 cells, driven by either peptide structure or cell-specific system bias (Figure 2G). Indeed, noted differences in phosphodiesterase (PDE) isoform expression across tissues, and a potentially unique interaction between specific GLP-1R agonists and these distinct PDE landscapes, may contribute to the variable cAMP responses observed across cell types [20,21]. Together these agonists exhibit similar intracellular pharmacodynamic profiles, with no significant differences elicited by C-terminal E2 conjugation relative to the GLP-1-CEX backbone on key GLP-1R signaling and internalization endpoints across cell types.

**Table 1:** Maximal (Emax) drug effects and potencies (pEC50, EC50) at the GLP-1R across HEK293T cell, mouse β-cell MIN6 cell, and mouse hypothalamic neuron CLU177 cell models. Dose-responses of each agonist were derived from the iAUC of a temporal response for each concentration/agonist. Emax, pEC50, and EC50 values were generated from dose–response values fitted to sigmoidal curves using a three-parameter non-linear regression. Emax is expressed as a % of the maximum response of GLP-1 (7-36)amide. The EC50 is the molar concentration that produced half of the maximal response. The pEC50 is the negative logarithm of the EC50. Values are provided for Gαs recruitment, cAMP production, Gαq recruitment, GLP-1R internalization, and β-arrestin 2 recruitment. Statistical significance was determined using one-way ANOVA utilizing Tukey's multiple comparisons correction. ∗ marks p ≤ 0.05 vs GLP-1 (7-36)amide. † marks p ≤ 0.05 vs GLP-1-CEX. N/A refers to agonists where curve fitting was incomplete.

		HEK293T			Min6			CLU177		
		Emax	pEC50	EC50 (nM)	Emax	pEC50	EC50 (nM)	Emax	pEC50	EC50 (nM)
G⍺s recruitment	**GLP-1 (7-36)amide**	100 ± 3.1	7.0 ± 0.1	97.08	100 ± 9.1	6.8 ± 0.2	162.7	100 ± 10.2	7.0 ± 0.2	96.49
	**GLP-1-CEX**	90.9 ± 4.9	7.3 ± 0.1	47.98	99.9 ± 10.8	6.8 ± 0.2	154.2	101.5 ± 8.4	7.0 ± 0.2	94.18
	**GLP-1-CEX/E2**	94.2 ± 5.2	7.4 ± 0.1	44.55	95.4 ± 7.7	6.8 ± 0.2	143.4	103.1 ± 5.7	7.2 ± 0.1	62.17
	**E2**	N/A	N/A	N/A	N/A	N/A	N/A	N/A	N/A	N/A
cAMP production	**GLP-1 (7-36)amide**	100 ± 3.1	7.9 ± 0.1	13.95	100 ± 1.2	9.8 ± 0.04	0.161	100 ± 5.3	9.7 ± 0.2	0.223
	**GLP-1-CEX**	98.7 ± 3.1	7.7 ± 0.1	17.97	95.9 ± 3.1	8.6 ± 0.1^a^	2,446	98.7 ± 4.1	10.9 ± 0.2†	0.012
	**GLP-1-CEX/E2**	111.1 ± 5.3	7.8 ± 0.1	16.76	99.1 ± 4.0	9.0 ± 0.1^a^^,^^b^	0.963	106.2 ± 6.2	10.5 ± 0.2	0.035
	**E2**	N/A	N/A	N/A	N/A	N/A	N/A	N/A	N/A	N/A
G⍺q recruitment	**GLP-1 (7-36)amide**	100 ± 6.4	7.2 ± 0.1	56.81	100 ± 13.2	6.4 ± 0.2	384.7			
	**GLP-1-CEX**	95.7 ± 3.9	7.4 ± 0.1	38.18	82.1 ± 13.3	6.6 ± 0.2	272.7			
	**GLP-1-CEX/E2**	94.0 ± 9.0	7.3 ± 0.2	47.77	76.7 ± 12.2	6.6 ± 0.3	244.6			
	**E2**	N/A	N/A	N/A	N/A	N/A	N/A			
GLP-1R internalization	**GLP-1 (7-36)amide**	100 ± 4.1	7.6 ± 0.1	23.91	100 ± 11.0	6.6 ± 0.2	226.6	100 ± 18.5	6.6 ± 0.3	245.4
	**GLP-1-CEX**	91.0 ± 1.5	8.0 ± 0.04^a^	10.13	100.5 ± 10.1	6.7 ± 0.2	190.8	104.0 ± 16.0	6.5 ± 0.2	282.9
	**GLP-1-CEX/E2**	90.9 ± 1.5	8.3 ± 0.1^a^	5,626	98.6 ± 9.3	6.8 ± 0.2	157.6	97.4 ± 14.8	6.8 ± 0.3	142.2
	**E2**	N/A	N/A	N/A	N/A	N/A	N/A	N/A	N/A	N/A
βarr2 recruitment	**GLP-1 (7-36)amide**	100 ± 2.4	7.0 ± 0.1	95.2	100 ± 5.8	7.2 ± 0.2	57.99	100 ± 9.1	7.0 ± 0.2	110.0
	**GLP-1-CEX**	93.6 ± 1.5	7.3 ± 0.04	50.1	103.7 ± 6.9	7.1 ± 0.2	84.1	107.3 ± 13.6	6.6 ± 0.2	275.6
	**GLP-1-CEX/E2**	100.2 ± 2.6	7.5 ± 0.1^a^	29.5	99.0 ± 4.9	7.4 ± 0.2	39.18	95.9 ± 4.1	6.8 ± 0.1	162.3
	**E2**	N/A	N/A	N/A	N/A	N/A	N/A	N/A	N/A	N/A

N/A refers to agonists where there was incomplete curve fitting or no significantly different result to 0 at high concentrations.

a Marks p ≤ 0.05 vs GLP-1 (7−36)amide.

b Marks p ≤ 0.05 vs GLP-1-CEX.

### 2.2 GLP-1-CEX/E2 induces receptor trafficking to LAMP1 positive lysosomal compartments via RAB7+ late endosomal routes

**Figure 3:**
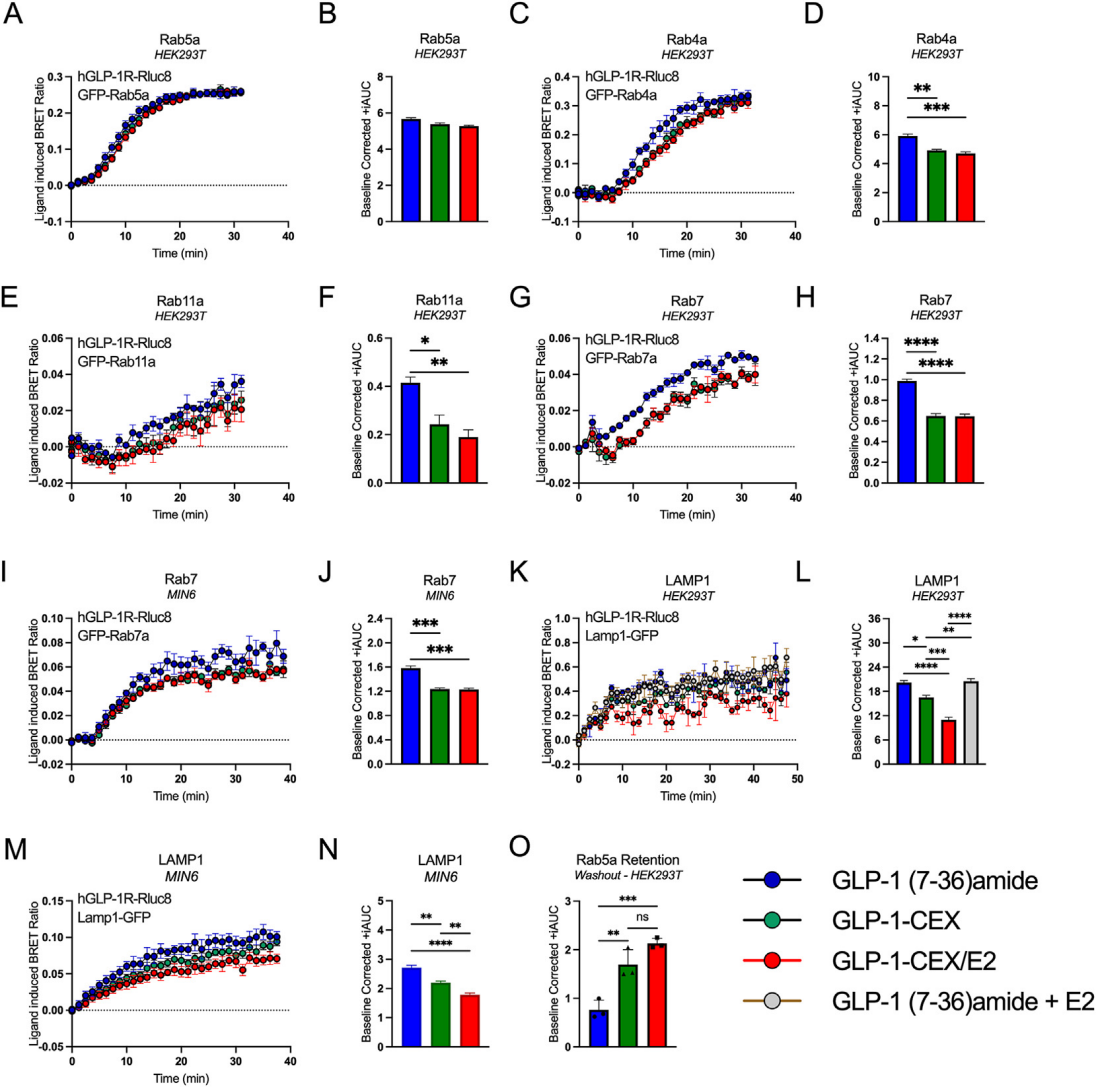
Ligand-induced GLP-1R trafficking via early and late endosomal routes to endo-lysosomal and lysosomal compartments. Colocalizations of GLP-1R-Rluc8 with GFP-tagged compartments were recorded using BRET. Data within Figures A–H, K–L, and O were performed in HEK293T cells; Figures I–J and M–N were performed in the mouse β-cell MIN6 cell model. Temporal, and associated baseline-corrected AUC, for colocalization of GLP-1R with **(A**–**B)** Rab5a^+^ early endosomes; **(C**–**D)** fast-recycling Rab4a^+^ endosomes; **(E**–**F)** slow-recycling Rab11a^+^ endosomes; **(G**–**H)** Rab7^+^ late endosomes; **(I**–**J)** Rab7^+^ late endosomes within MIN6 cells; (**K-L)** LAMP1^+^ lysosomal compartments within HEK293T cells; and **(M**–**N)** LAMP1^+^ lysosomal compartments within MIN6 cells. **(O)** Colocalization of GLP-1R with Rab5a^+^ early endosomes following ligand stimulation and subsequent washout. Agonists were used at 1 μM and at least three independent experiments were performed with multiple technical replicates per group. For +iAUC graphs expressed as mean ± SEM for each agonist, ∗p ≤ 0.05, ∗∗p ≤ 0.01, and ∗∗∗p ≤ 0.001, ∗∗∗∗p ≤ 0.0001 using one-way ANOVA with Tukey's multiple comparisons correction.

**Supplementary Figure 2:**
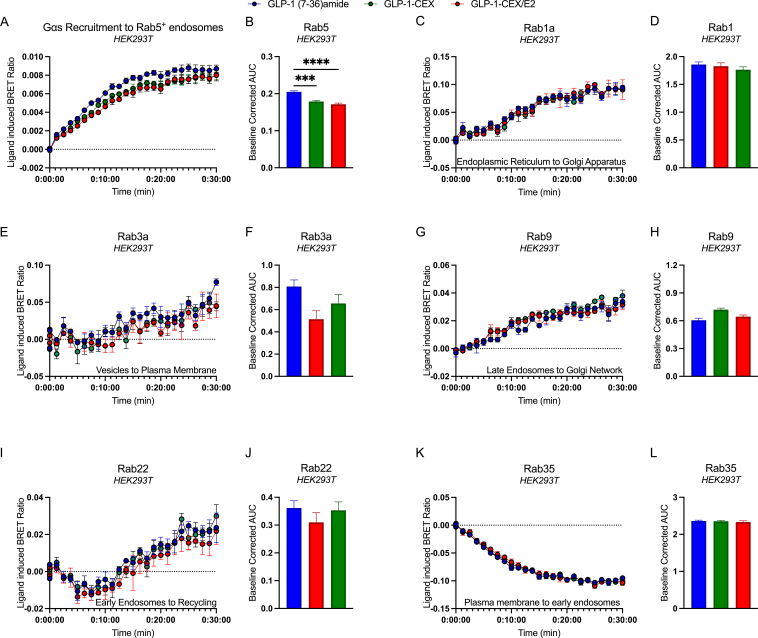
(A) Temporal and **(B)** AUC quantifications for ligand-induced Nluc-mGα_s_ recruitment to GLP-1R^+^/Rab5-Venus^+^ endosomes. Temporal and AUC quantifications for ligand-induced GLP-1R-Rluc8 colocalization with endosomes expressing **(C**–**D)** Rab1-Venus^+^, **(E**–**F)** Rab3a-eGFP, **(G**–**H)** Rab9-Venus, **(I**–**J)** Rab22-eGFP, and **(K**–**L)** Rab35-eGFP. Experiments were performed in HEK293T cells using 3 biological replicates with at least two technical replicates per agonist. ∗p ≤ 0.05, ∗∗p ≤ 0.01, ∗∗∗p ≤ 0.001, ∗∗∗∗p ≤ 0.0001 using an unpaired two-tailed t test, or a one-way ANOVA with Tukey correction for multiple comparisons.

Following ligand binding to plasma membrane-localized GPCRs, their subsequent internalization into intracellular environments and redistribution across endolysosomal compartments represent potentially key therapeutic targets [[22], [23], [24]]. Post-internalization, GPCRs often colocalize into early endosomes, marked by Ras-related protein Rab-5 (Rab5) [25,26]. The GLP-1R has previously demonstrated differential ligand-induced colocalization into Rab5^+^ early endosomes, particularly when comparing GLP-1R mono-agonist semaglutide and the GLP-1R/Glucose-dependent insulinotropic polypeptide receptor (GIPR) dual-agonist tirzepatide [24,27]. Hence, we tested GLP-1 (7-36)amide, GLP-1-CEX, and GLP-1-CEX/E2 for their ability to stimulate GLP-1R colocalization into Rab5^+^ compartments, as well as multiple downstream endolysosomal compartments. Like the previously observed GLP-1R internalization dynamics (Figure 2E,J, N), GLP-1-CEX/E2 induces GLP-1R colocalization into Rab5^+^ early endosomes with only minor differences in temporal dynamics and area under the curve (AUC) relative to GLP-1 (7-36)amide and GLP-1-CEX (Figure 3A–B). The continuation of GLP-1R Gαs recruitment within Rab5^+^ early endosomes has been suggested to contribute to total cAMP generation [[27], [28], [29]]. Here, GLP-1-CEX and GLP-1-CEX/E2 do not differ in their ability to recruit Gαs into GLP-1R^+^ Rab5^+^ endosomes, suggesting C-terminal E2 conjugation to not influence compartment-specific Gαs coupling capacity; however Gαs recruitment was slightly but significantly decreased in a CEX-specific manner relative to GLP-1 (7-36)amide (Supplementary Figs. 2A–B). Following early endosome colocalization, the GLP-1R traffics within various endosomal compartments marked by Rab GTPases Rab4 (fast recycling endosomes), Rab11a (slow recycling endosomes), and Rab7 (late endosomes). In Rab4^+^ fast recycling endosomes, both GLP-1-CEX and GLP-1-CEX/E2 stimulate maximal GLP-1R colocalization, albeit with slightly slower kinetics relative to GLP-1 (7-36)amide, resulting in small but significant CEX-specific decreases in the AUC (Figure 3C–D). GLP-1-CEX and GLP-1-CEX/E2 exhibit similarly delayed CEX-specific colocalization dynamics with Rab11a^+^ slow recycling endosomes, exhibiting significant decreases in the AUC relative to GLP-1 (7-36)amide (Figure 3E–F). In Rab7^+^ late endosomes, both CEX-containing peptides, GLP-1-CEX and GLP-1-CEX/E2, show reduced maximal GLP-1R colocalization and kinetics relative to GLP-1 (7-36)amide, resulting in significant reductions in total AUC (Figure 3G–H). This reduction at Rab7 is replicated, though less pronounced, in MIN6 cells expressing the hGLP-1R (Figure 3I–J). After Rab7^+^ endosomal colocalization, canonical GLP-1R trafficking is directed into lysosomes. Consistent with the previous ligand-induced Rab7 colocalization dynamics, GLP-1R colocalization into LAMP1^+^ lysosomes is also significantly reduced with GLP-1-CEX and GLP-1-CEX/E2 relative to GLP-1 (7-36)amide, suggesting a likely ligand structure-driven phenomenon (Figure 3K-L). Relative to the GLP-1-CEX backbone, however, GLP-1-CEX/E2 exhibits a further decrease in LAMP1^+^ lysosomal colocalization, suggesting a possible influence of structural E2 conjugation, or alternatively, intracellular E2 action (Figure 3K-L). The lack of a similar reduction in GLP-1R colocalization when free E2 (1 μM) is co-administered with GLP-1 (7-36)amide indicates that the structural conjugation of E2 to the GLP-1-CEX backbone specifically affects GLP-1R lysosomal colocalization, even without observable differences in earlier steps of the endolysosomal pathway. These differential lysosomal colocalization dynamics between GLP-1-CEX and GLP-1-CEX/E2 were also observed in hGLP-1R^+^ MIN6 cells (Figure 3M−N). To better understand the CEX-specific decreases observed in endosomal trafficking pathways downstream of Rab5^+^ early endosomes, GLP-1R retention within Rab5^+^ early endosomes following immediate ligand washout was assessed. This approach theoretically limits new GLP-1R influx into early endosomes, allowing for a clearer resolution of GLP-1R efflux. Both GLP-1-CEX and GLP-1-CEX/E2 retain more GLP-1R within Rab5^+^ early endosomes than GLP-1 (7-36)amide (Figure 3O). This CEX-specific retention likely accounts for the observed decreases in trafficking within more quantitatively sensitive endosomal compartments, including those positive for Rab4, Rab11, Rab7, and Lamp1. Other endosomal compartments were assessed for ligand-stimulated GLP-1R colocalization, including Rab1^+^, Rab3^+^, Rab9^+^, Rab22^+^, and Rab35^+^, of which are involved in various facets of receptor synthesis, recycling, and early endosome maturation. However, no obvious differences between GLP-1 (7-36)amide, GLP-1-CEX, and GLP-1-CEX/E2 were identified within any of these endosomal compartments (Supplementary Fig. 2C-L).

In summary, GLP-1-CEX and GLP-1-CEX/E2 sort via the GLP-1R into Rab5^+^ early endosomes, Rab4^+^ fast recycling endosomes, and Rab11^+^ slow recycling endosomes with general similarity to that of GLP-1 (7-36)amide, albeit with slightly slower kinetics. In Rab7^+^ late endosomes and LAMP1^+^ lysosomes, GLP-1-CEX and GLP-1-CEX/E2 exhibit reduced GLP-1R colocalization relative to GLP-1 (7-36)amide, with this reduction slightly exaggerated at the lysosome by C-terminal E2 conjugation.

### 2.3 The E2 moiety of GLP-1-CEX/E2 co-internalizes with the GLP-1R within the intracellular space

**Figure 4:**
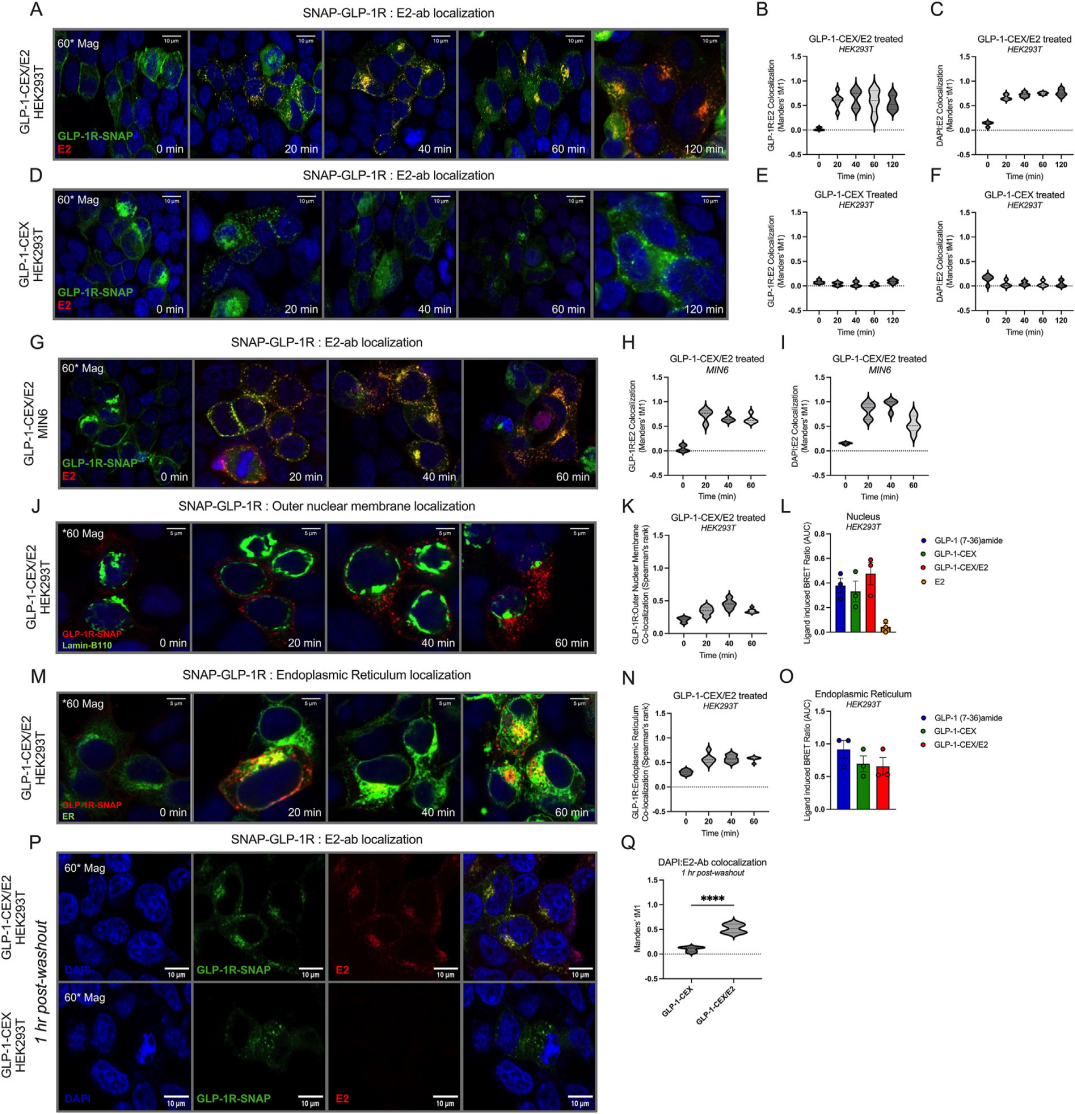
ICC images depicting the time-dependent co-internalization of the GLP-1-CEX/E2 ligand with the SNAP-GLP-1R, and trafficking to the nuclear membrane and ER. **(A)** Visualization and **(B)** Manders' tM1 of SNAP-GLP-1R colocalization with a primary antibody specific to the unconjugated end 17β-estradiol (E2-ab), and **(C)** Manders' tM1 of the DAPI stain with the E2-ab, in SNAP-GLP-1R overexpressing HEK293T cells treated with GLP-1-CEX/E2 (1 μM), and subsequently fixed with 4% PFA at varying time-points between 2 h. **(D)** Visualization and Manders' tM1 of **(E)** SNAP-GLP-1R colocalization with E2-ab and **(F)** DAPI colocalization with E2-ab in HEK293T cells treated with GLP-1-CEX (1 μM). **(G)** Visualization and Manders' tM1 of GLP-1-CEX/E2-induced (1 μM) trafficking and colocalization of **(H)** SNAP-GLP-1R with E2-ab and **(I)** DAPI colocalization with E2-ab in MIN6 cells over 60 min. **(J)** Visualization and **(K)** Spearman's rank correlation of ligand-induced (1 μM) trafficking and colocalization of SNAP-GLP-1R with Lamin-B110-mNeongreen, an outer nuclear membrane marker, over the course of 60 min. **(L)** Ligand-induced BRET ratio AUC of GLP-1R-GFP with NLS-Nluc following ligand stimulation over 30 min. **(M)** Visualization and **(N)** Spearman's rank correlation of ligand-induced (1 μM) trafficking and colocalization of SNAP-GLP-1R with ER-mNeongreen over the course of 60 min. **(O)** Ligand-induced BRET ratio AUC of GLP-1R-Rluc8 with ER-mNeongreen following ligand stimulation over 30 min. **(P**–**Q)** E2-ab colocalization with SNAP-GLP-1R and DAPI following stimulation of GLP-1-CEX/E2 (1 μM) and 1 h washout. +iAUC graphs expressed as mean ± SEM for each agonist. Agonists were provided at 1 μM, experiments were performed independently three times. Cell counts for each confocal image analysis are between 5 and 13 cells per time point.

**Supplementary Figure 3:**
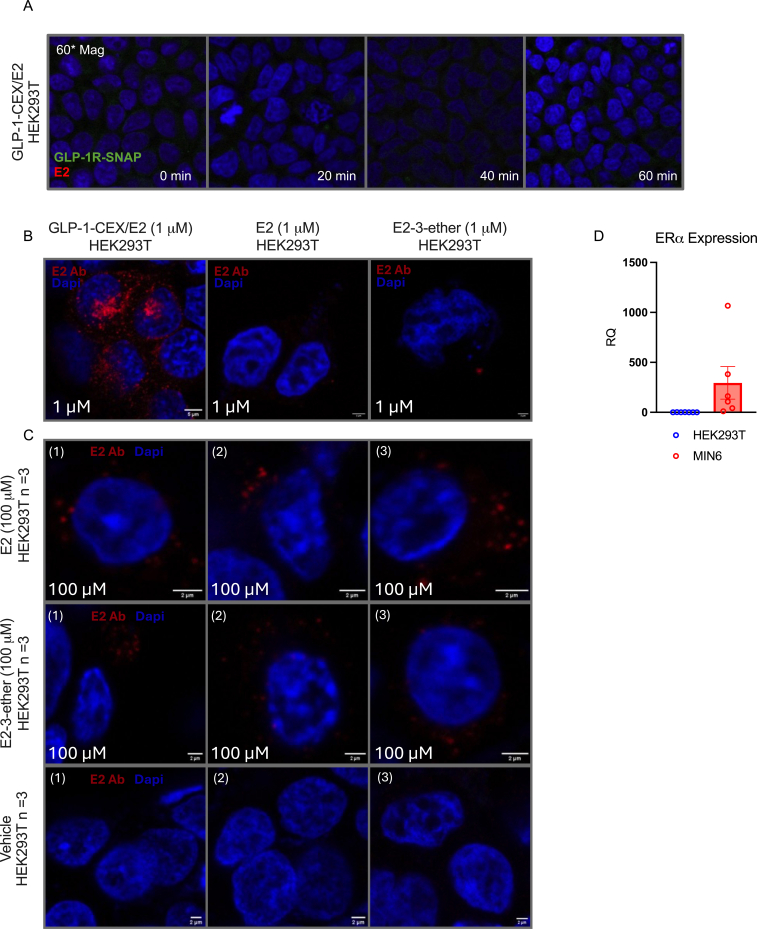
(A) Non transfected HEK293T cells treated with GLP-1-CEX/E2, fixed every 20 min and stained with DAPI and E2-ab. **(B)** GLP-1R^+^ HEK293T cells treated with GLP-1-CEX/E2, E2, and E2-3-ether at 1 μM for 1 h and stained with DAPI and E2-ab. **(C)** GLP-1R^+^ HEK293T cells treated with E2 and E2-3-ether at 100 μM for 1 h and stained with DAPI and E2-ab, in triplicates. **(D)** RQ values for ERα in HEK293T cells and MIN6 cells. ∗p ≤ 0.05, ∗∗p ≤ 0.01, ∗∗∗p ≤ 0.001, ∗∗∗∗p ≤ 0.0001 using an unpaired two-tailed t test, or a one-way ANOVA with Tukey correction for multiple comparisons.

**Supplementary Figure 4:**
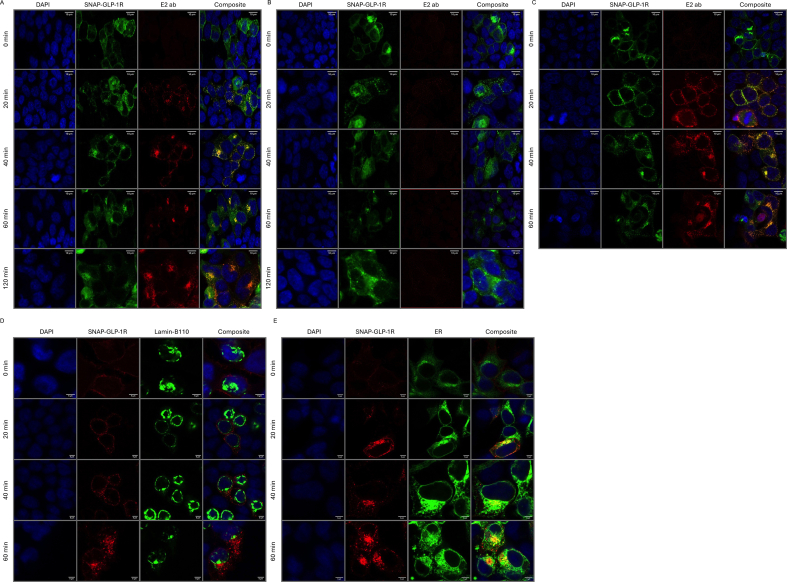
Confocal images with individual channels visualized from Figure 4: **(A)** GLP-1-CEX/E2 (1 μM) treatment in SNAP-GLP-1R^+^ HEK293T cells over 120 min; **(B)** GLP-1-CEX (1 μM) treatment in SNAP-GLP-1R^+^ HEK293T cells over 120 min; **(C)** GLP-1-CEX/E2 (1 μM) treatment in SNAP-GLP-1R^+^ MIN6 cells over 60 min; **(D)** GLP-1-CEX/E2 (1 μM) treatment in SNAP-GLP-1R^+^ mEmerald-LaminB1-10^+^ HEK293T cells over 60 min; **(E)** GLP-1-CEX/E2 (1 μM) treatment in SNAP-GLP-1R^+^ ER-mNeonGreen^+^ HEK293T cells over 60 min.

We next visualized and assessed the intracellular localization of the E2 moiety of GLP-1-CEX/E2 to better understand liberation dynamics. We utilized SNAP-GLP-1R^+^ HEK293T cells and immunohistochemical fluorescent labelling of the unconjugated hydroxyl group-containing position 17 of estradiol, to simultaneously track the positions of GLP-1R and the E2 moiety of GLP-1-CEX/E2 over time. Following plasma membrane-specific GLP-1R labelling with a membrane-impermeable SNAP-tag substrate, treatment with GLP-1-CEX/E2 (1 μM) led to the intracellular colocalization of SNAP-GLP-1R and E2-ab, as well as E2-ab colocalization with the nuclear stain DAPI, at 20, 40, 60, and 120 min (Figure 4A–C). This indicates a substantial accumulation of intracellular GLP-1R bound to GLP-1-CEX/E2, and overlap of the E2 antibody with the nucleus, over time. In HEK293T cells lacking SNAP-GLP-1R expression, GLP-1-CEX/E2 did not evoke any intracellular E2-ab accumulation, demonstrating the necessity of GLP-1R endocytosis for the intracellular accumulation of the E2 moiety, as well as an apparent lack of endogenous E2 content (Supplementary Fig. 3A). Further, following 1 h incubation in GLP-1R^+^ HEK293T cells, GLP-1-CEX/E2 (1 μM) facilitates greater intracellular accumulation of E2-ab signal relative to 1 μM and 100 μM of free E2 and E2-3-ether (Supplementary Figs. 3B–C). However interpretation is limited due to the physiochemical differences between free E2 and the GLP-1-CEX-bound E2, despite the E2-ab being able to engage the epitope of both molecules equally. In SNAP-GLP-1R^+^ HEK293T cells, administration of the unconjugated GLP-1-CEX backbone led to substantial intracellular GLP-1R punctate formation without E2 immunoreactivity (Figure 4D–F). SNAP-GLP-1R^+^ MIN6 cells, which prominently express estrogen receptor alpha (ERα) (Supplementary Fig. 3D), exhibit similar GLP-1R/E2-ab and DAPI/E2-ab colocalization patterns as seen in HEK293T cells when treated with GLP-1-CEX/E2 (Figure 4A–C; Figure 4G–I). GLP-1-CEX/E2 localized GLP-1R to Lamin-B110, an outer nuclear membrane marker and surrogate for the perinuclear space, with a time-dependent capacity similar to both GLP-1 (7-36)amide and GLP-1-CEX, as observed in both confocal analysis and BRET (Figure 4J-L). This suggests that the proximal localization of ligand-bound GLP-1R to the nucleus is an innate phenomenon. The conjugate also time-dependently co-localized GLP-1R with a GFP-tagged KDEL sequence, marking the endoplasmic reticulum, without significant difference from controls, suggesting a lack of distinct bias in spatial compartmentalization between agonists (Figure 4M−O). Lastly, following treatment with GLP-1-CEX/E2 and a 1 h washout incubation, the E2-ab continued to colocalize with the nuclear DAPI stain, suggesting sustained nuclear proximity of the E2 moiety even after ligand input was discontinued (Figure 4P-Q). All channels for each visual representation are available (Supplementary Figs. 4A–E).

In summary, these data demonstrate significant GLP-1-CEX/E2-stimulated GLP-1R-dependent internalization of the E2 moiety, colocalization of GLP-1R with the nuclear outer membrane and endoplasmic reticulum, and an indication of potential E2 moiety entry into the nuclear space.

### 2.4 The amplitude of GLP-1-CEX/E2 engagement with ER activation is dependent on lysosomal acidification and potency of E2-3-ether at ERα

**Figure 5:**
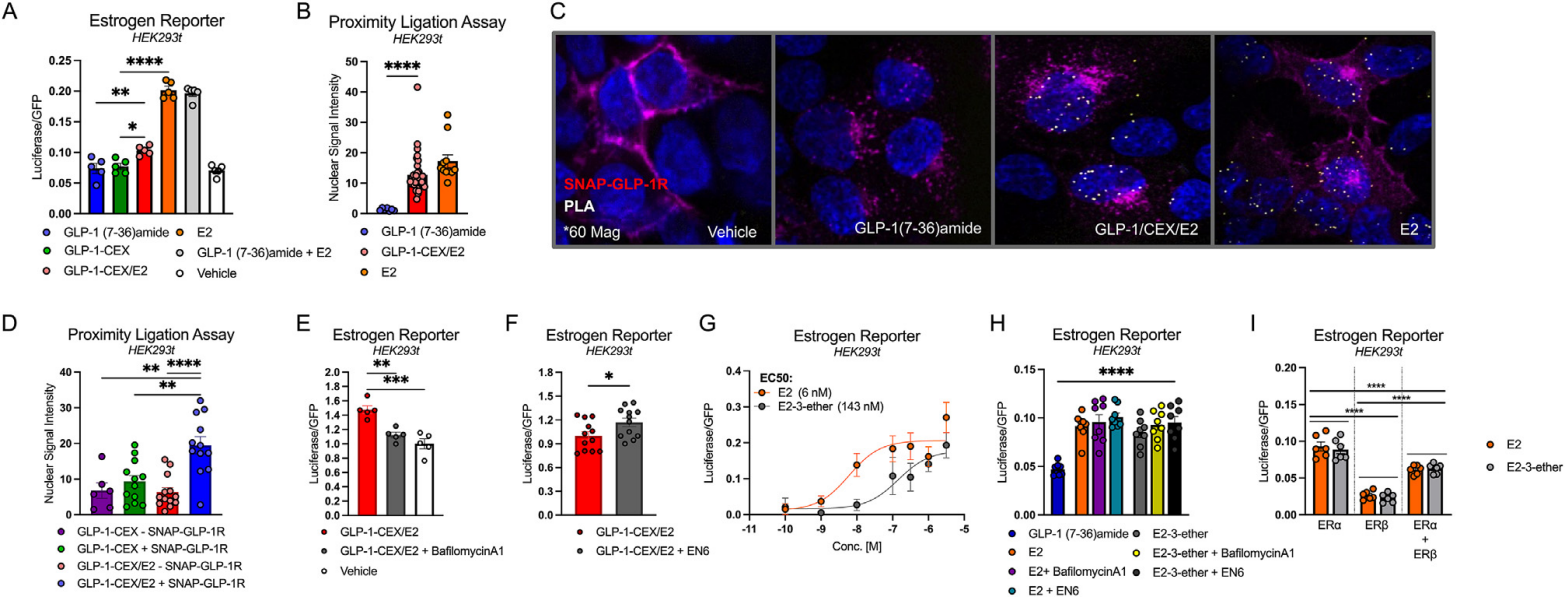
GLP-1-CEX/E2 and E2 agonist-stimulated signaling in model HEK293T cells. **(A)** Estrogen response element (ERE) luciferase reporter activity following 24hr incubation ligand-incubation in HEK293T cells overexpressing hGLP-1R and ERα. Proximity Ligation Assay (PLA) between ERα and SP-1 following ligand administration was quantified via **(B)** average nuclear PLA signal intensity and **(C)** visualized for each condition in HEK293T cells overexpressing hGLP-1R and ERα. PLA quantification with ERα and SP-1 **(D)** in HEK293T cells either expressing or lacking the GLP-1R, following incubation with either GLP-1-CEX or GLP-1-CEX/E2. **(E)** ERE luciferase reporter of GLP-1-CEX/E2 vs co-administration of GLP-1-CEX/E2 with VATPase inhibitor bafilomycinA1 (5 μM) following 24 h incubation. **(F)** ERE luciferase reporter of GLP-1-CEX/E2 vs co-administration of GLP-1-CEX/E2 with VATPase activity enhancer EN6 (50 μM) over 24 h incubation. **(G)** Vehicle-subtracted ERE reporter dose–response to 24 h E2 and E2-3-ether incubation in ERα and GFP expressing HEK293T cells. **(H)** Vehicle-subtracted ERE reporter activity following 24 h incubation of either E2 or E2-3-ether with bafilomycinA1 (5 μM) or EN6 (50 μM) in HEK293T cells. **(I)** Vehicle-subtracted ERE reporter activity following 24 h incubation of either E2 or E2-3-ether in HEK293T cells overexpressing either ERα, ERβ, or both. GLP-1-based peptides and E2 agonists were used at 1 μM apart from in (F). Three independent experiments were performed with at least two technical replicates per group. ∗p ≤ 0.05, ∗∗p ≤ 0.01, and ∗∗∗p ≤ 0.001, ∗∗∗∗p ≤ 0.0001 using an unpaired two-tailed t test, or a one-way ANOVA with Tukey correction for multiple comparisons.

To further validate nuclear functionality of the E2 moiety of GLP-1-CEX/E2, we utilized an estrogen response element (ERE) luciferase reporter in HEK293T cells overexpressing hGLP-1R and ERα. We observed a significant increase in luciferase activity with GLP-1-CEX/E2 (1 μM) relative to GLP-1 (7-36)amide and GLP-1-CEX, indicating intact transcriptional functionality of the potentially liberated E2 moiety (Figure 5A). However, this increase was significantly lower than that observed with the passively diffusing free E2 (1 μM) (Figure 5A), suggesting potential limitations on GLP-1-CEX/E2 that may be due to the kinetics of GLP-1R-mediated entry and endolysosomal liberation of the E2 moiety, or potency of the liberated E2 moiety at ERα. Equimolar co-administration of GLP-1 (7-36)amide and E2 does not negatively affect the ERE response, indicating ERE activity to be independent of GLP-1R signaling (Figure 5A). We complemented these findings with proximity ligation assays (PLA) in SNAP-GLP-1R^+^ HEK293T, showing that both GLP-1-CEX/E2 and free E2 significantly increase the proximal localization of ERα and the nucleus-localized transcription factor specificity protein-1 (SP-1) relative to GLP-1 (7-36)amide (Figure 5B–C). In line with GLP-1R-dependent intracellular accumulation of the E2 moiety of GLP-1-CEX/E2 (Figure 4A–C; Supplementary Fig. 3A), we further demonstrate that GLP-1R expression and E2 conjugation are required for mediating GLP-1-CEX/E2-stimulated increases in proximal localization of ERα and SP-1 within the nucleus (Figure 5D).

**Supplementary Figure 5:**
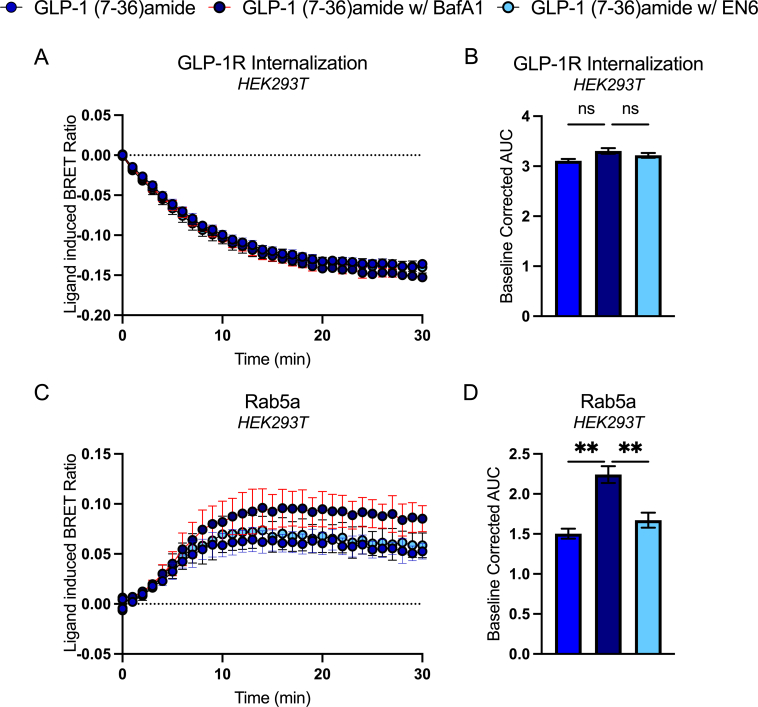
Temporal and AUC quantifications of GLP-1R **(A**–**B)** internalization and **(C**–**D)** Rab5^+^ early endosome colocalization, in response to GLP-1 (7-36)amide (1 μM) when pre- and co-treated with bafilomycinA1 (5 μM) or EN6 (50 μM) in GLP-1R^+^ HEK293T cells.

A limiting factor in ERE activation by GLP-1-CEX/E2 may be the rate of E2 moiety liberation, which is suggested to depend on lysosomal localization and acidification [13,16]. We tested the effect of inhibiting lysosomal acidification using the lysosomal VATPase inhibitor bafilomycinA1 (5 μM), which significantly decreased GLP-1-CEX/E2-stimulated ERE activity to that comparable of the vehicle (Figure 5E). However, bafilomycinA1 has been associated with off-target reductions in endolysosomal trafficking in bulk [30], suggesting that the reduction in ERE activity might be due to reduced GLP-1R endocytosis flux and not reduced lysosomal acidification. We validate this claim by showing bafilomycinA1 pre/co-treatment significantly increases GLP-1R retention in Rab5^+^ early endosomes without affecting receptor internalization, suggesting impaired downstream endosomal trafficking (Supplementary Figs. 5A–B). To investigate more precisely, we pre- and co-treated cells with the small molecule lysosomal VATPase activator EN6 (50 μM) to enhance lysosomal acidification, which significantly potentiated the ERE activity of GLP-1-CEX/E2 without altering endosomal trafficking dynamics, highlighting the role of lysosomal acidification in E2 moiety liberation (Figure 5F; Supplementary Figs. 5C–D). The amide/ether linker connecting GLP-1-CEX to E2 is designed to be sensitive to lysosomal proteolytic cleavage, potentially yielding the metabolite E2-3-ether rather than E2 itself (Figure 1B–C) [7]. Both E2 and E2-3-ether dose-dependently stimulate ERE activity in HEK293T cells overexpressing ERα, albeit E2-3-ether less potently (Figure 5G). BafilomycinA1 and EN6 did not have off-target effects on stimulated ERα activation by E2 or E2-3-ether, therefore ERα activity is likely not indirectly influenced by the lysosomal acidification state (Figure 5H). Further, ERE activity may involve ERα homodimers, ERβ homodimers, and ERα/ERβ heterodimers. However, E2 and E2-3-ether do not show bias in engaging specific receptor subtypes at saturating doses, with ERα homodimerization predominating as the most prominent facilitator of ERE activity for both ligands (Figure 5I).

Together, these data suggest that GLP-1-CEX/E2-induced ERα activity relies on both GLP-1R expression and lysosomal acidification. Due to the amide/ether linker strategy, lysosomal cleavage of the E2 moiety from the GLP-1-CEX backbone is projected to result in E2-3-ether rather than E2 itself. Nonetheless, both E2 and E2-3-ether dose-dependently increase ERE activity without biases at either estrogen receptor subtype.

### 2.5 GLP-1-CEX/E2 leads to intracellular increases in estradiol, estrone-3-sulfate, and estrone likely originating from E2-3-ether conversion into estrone-3-sulfate

**Figure 6:**
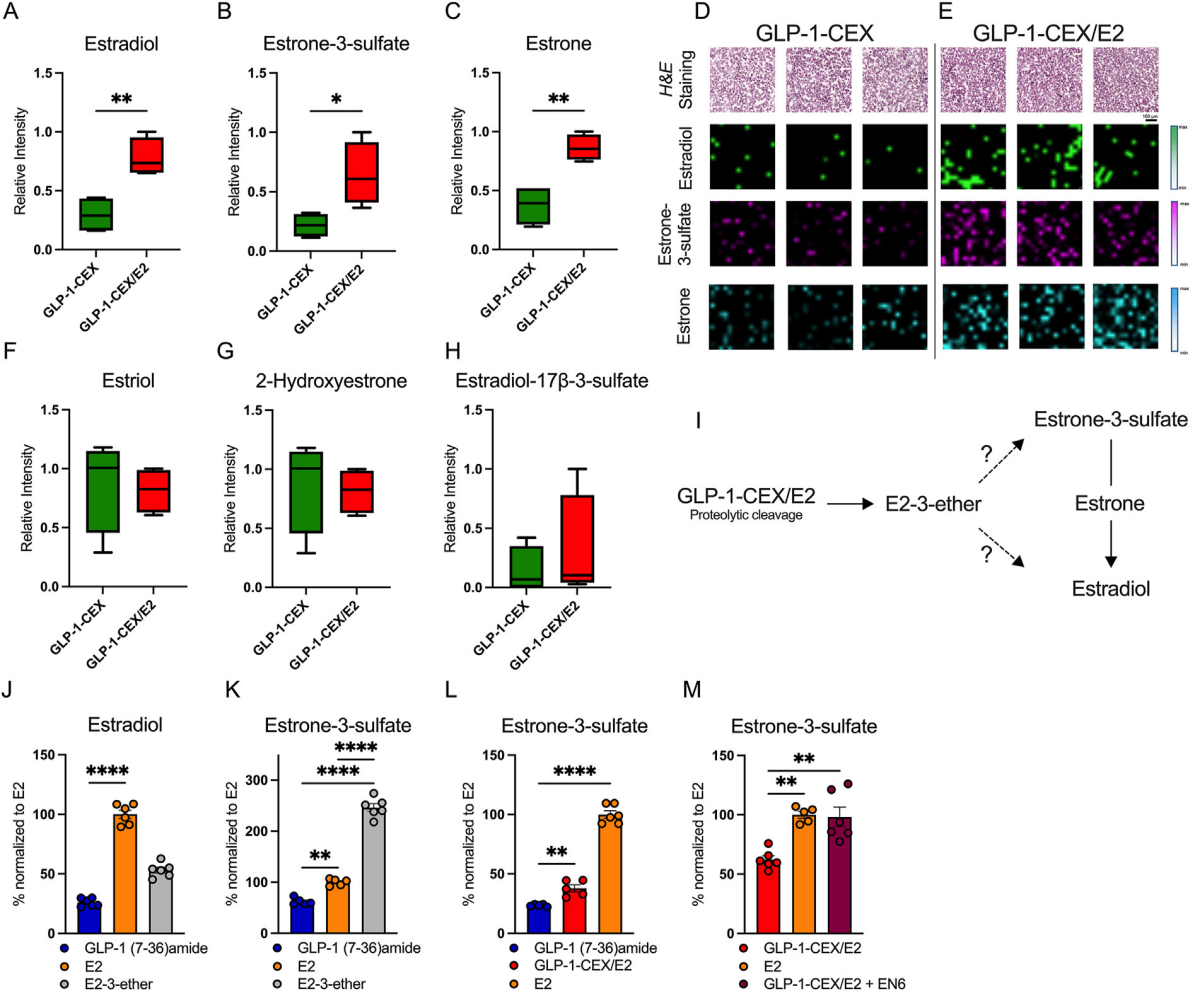
GLP-1-CEX and GLP-1-CEX/E2 differentially affect E2 and estrogenic metabolite abundance following incubation in HEK293T cells. MALDI-IMS recording of relative changes in **(A)** 17β-estradiol, **(B)** estrone-3-sulfate, and **(C)** estrone in GLP-1-CEX or GLP-1-CEX/E2 treated cell pellet slices. Triplicate visual representation of 17β-estradiol, estrone-3-sulfate or estrone in **(D)** GLP-1-CEX or **(E)** GLP-1-CEX/E2 treated cell pellet slices. Detected levels of **(F)** Estriol, **(G)** 2-hydroxyestrone, and **(H)** estradiol-17β-3-sulfate using MALDI-IMS. **(I)** Schematic indicating the hypothesized metabolic routes of the liberated E2 derivative. **(J)** 17β-estradiol-specific ELISA quantification following 2 h treatment with GLP-1 (7-36)amide, E2, or E2-3-ether at 1 μM in HEK293T cells. **(K)** Estrone-3-sulfate-specific ELISA quantification following 2 h treatment with GLP-1 (7-36)amide, E2, or E2-3-ether at 1 μM in HEK293T cells. **(L)** Estrone-3-sulfate-specific ELISA quantification following 2 h treatment with GLP-1 (7-36)amide, GLP-1-CEX/E2, or E2 at 1 μM in HEK293T cells. **(M)** Estrone-3-sulfate-specific ELISA quantification following 2 h treatment with GLP-1-CEX/E2 (1 μM), E2 (1 μM), or co-treatment with GLP-1-CEX/E2 (1 μM) and EN6 (50 μM) in HEK293T cells. GLP-based peptides and E2 agonists were used at 1 μM, at least three independent experiments were performed with two technical replicates per group. ∗p ≤ 0.05, ∗∗p ≤ 0.01, ∗∗∗p ≤ 0.001, ∗∗∗∗p ≤ 0.0001 using an unpaired two-tailed t test, or a one-way ANOVA with Tukey correction for multiple comparisons.

**Supplementary Figure 6:**
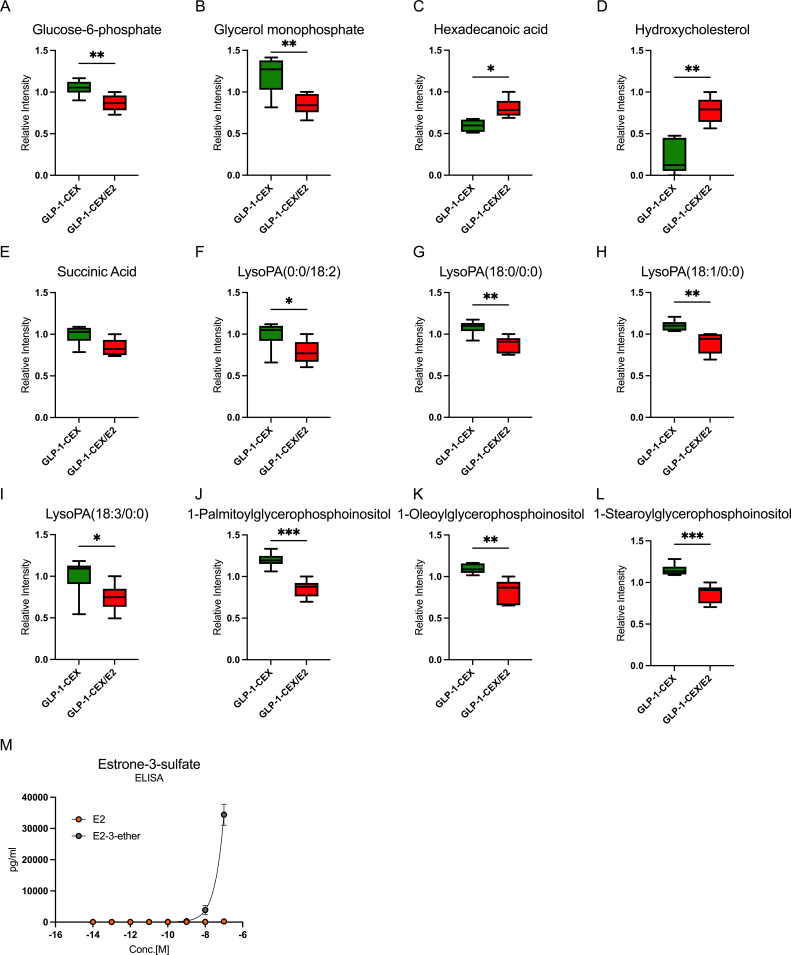
(A–**L)** GLP-1R^+^ HEK293T pellet slices treated with GLP-1-CEX or GLP-1-CEX/E2 (1 μM) evaluated by MALDI-IMS exhibit relative changes in multiple metabolites not directly categorized as estrogenic. **(M)** Estrone-3-sulfate-specific ELISA quantification evaluating assay non-specificity by direct dose–response application of E2 and E2-3-ether without cells. Dose response was carried out and analysed as in Figure 2. ∗p ≤ 0.05, ∗∗p ≤ 0.01, ∗∗∗p ≤ 0.001, ∗∗∗∗p ≤ 0.0001 using an unpaired two-tailed t test, or a one-way ANOVA with Tukey correction for multiple comparisons.

We found the predicted liberated metabolite E2-3-ether activates ERE transcription, though with substantially lower potency relative to E2 (Figure 5G). Given that GLP-1-CEX/E2 relies on a limited membrane-localized GLP-1R population for cellular entry of the conjugated E2 moiety, we explored whether enzymatic conversion of E2-3-ether to the more potent E2 may overcome these upstream GLP-1R quantitative limitations. Using matrix-assisted-laser-desorption-ionization imaging mass spectrometry (MALDI-IMS), we performed spatial metabolomics on a layer of HEK293T cells expressing hGLP-1R and treated with GLP-1-CEX/E2 (1 μM) or GLP-1-CEX (1 μM). This analysis revealed, for the first time *in vitro*, significant increases in native E2 following acute treatment of GLP-1-CEX/E2 over 2 h (Figure 6A, D-E). Additionally, we observed significant increases in estrone-3-sulfate (E1S) and estrone, both readily interconvertible metabolites of estradiol that exhibit minimal ERα affinity and therefore function as a reservoir to buffer against excess estradiol accumulation (Figure 6B–E) [[31], [32], [33], [34]]. However, we did not detect E2-3-ether or other metabolites related to the degradation or inactivation of E2, E1S, or estrone (Figure 6F–H). Interestingly, GLP-1-CEX/E2 treatment uniquely affected metabolites associated with glucose, fatty acid, and cholesterol metabolism (Supplementary Fig. 6A-L), which may lead to future investigation.

Despite E2-3-ether being the hypothesized product of amide/ether linker cleavage [7,13], it was not identified via MALDI-IMS. To investigate further, we assessed whether free E2-3-ether is metabolized into an alternative substrate (Figure 6I). Using an E2-specific ELISA, after 2 h of incubation with equimolar E2, E2-3-ether, or GLP-1 (7-36)amide in GLP-1R^+^ HEK293T cells, we found that E2-3-ether converts to E2 although at a limited rate, as indicated by an approximate 50% reduction relative to E2 (Figure 6J). Given the limited E2 presence following E2-3-ether administration and the observed GLP-1-CEX/E2 induced increase in estrone-3-sulfate (Figure 6B, D-E), we next assessed the possibility of E2-3-ether converting into estrone-3-sulfate as a limiting factor in estradiol conversion. Using an E1S-specific ELISA, free E2-3-ether significantly increased estrone-3-sulfate levels in GLP-1R^+^ HEK293T cells over 2 h, with twice the efficacy of free estradiol (Figure 6K). The increase in estrone-3-sulfate production by free E2-3-ether is unlikely due to off-target ELISA specificity, especially at the low concentrations expected to be present after multiple cell washes and subsequent lysis (Supplementary Fig. 6M). In this ELISA-based approach, GLP-1-CEX/E2 significantly, but subtly, increased estrone-3-sulfate production relative to GLP-1 (7-36)amide, though not to the extent of free E2 (Figure 6L). When the VATPase activity enhancer EN6 was co-administered with GLP-1-CEX/E2, surprisingly estrone-3-sulfate production matched that of freely diffusible E2, indicating that GLP-1R^+^ lysosome acidification strongly influences estrone-3-sulfate accumulation (Figure 6M). This suggests that the cleaved E2 moiety, likely E2-3-ether, may be metabolized into estrone-3-sulfate, making it a potentially sensitive marker for liberation from the GLP-1-CEX backbone.

Together, these data indicate that the estrogenic efficacy of GLP-1-CEX/E2 is mediated by GLP-1R internalization, lysosomal acidification, hydrolysis of the amide bond within the amide/ether linker, and the subsequent accumulation of inactive estrone-3-sulfate following the metabolic conversion of the potentially liberated E2-3-ether. This influx of estrone-3-sulfate may ultimately result in a limited shift towards estradiol conversion via estrone.

## 3 Discussion

GLP-1-CEX/E2 is an Aib2/Glu16/CEX-modified GLP-1 peptide covalently conjugated to the nuclear hormone E2 via an amide/ether linker [7]. This conjugate is designed to deliver its nuclear hormone receptor-acting cargo into GLP-1R^+^ cells, leading to parallel and synergistic GLP-1R and ERα/ERβ signaling, without off-target effects in GLP-1R lacking tissues. GLP-1-CEX/E2 synergistically enhances satiety, body weight control, glucoregulation, and β-cell resilience compared to GLP-1-CEX alone, while maintaining a similar safety profile [7,12,15,16]. This conjugate has paved the way for developing other peptide/small molecule conjugates for treating diabetes and obesity, such as GLP-1/dexamethasone [10], GLP-1/tesaglitazar [9], and GLP-1/MK-801 [11]. Despite the observed *in vivo* synergisms of these approaches, the mechanistic details of small molecule liberation from the GLP-1 peptide have remained understudied.

Leveraging live-cell bioluminescence resonance energy transfer (BRET) techniques, we first systematically examined the signaling and trafficking dynamics of the GLP-1R, using GLP-1-CEX/E2 as an exemplary GLP-1 conjugate. We identify that GLP-1R signaling, internalization, and endosomal trafficking in response to GLP-1-CEX/E2 were generally comparable to the unconjugated GLP-1 peptides. This indicates that the Aib2 and Glu16 substitutions, CEX addition, and C-terminal conjugation of E2, do not negatively impact GLP-1R-centric signaling and internalization processes. This supports the GLP-1-CEX C-terminal end as an appropriate site for conjugating small molecules for targeted delivery into GLP-1R^+^ cells. Of the primary differences, we identify Aib2/Glu16/CEX inclusion within the GLP-1 sequence reduces RAB7^+^ late endosomal trafficking relative GLP-1 (7-36)amide. Additionally, E2 conjugation to GLP-1-CEX further reduces LAMP1^+^ lysosomal accumulation relative to GLP-1-CEX. Lastly, subtle alterations in relative cAMP potency between ligands within different cell types highlight the need for further insight into cell-specific GLP-1R dynamics.

Importantly, using GLP-1-CEX/E2, we demonstrate GLP-1R-dependent internalization and subsequent proximal nuclear localization of the E2 moiety. We then show increased estrogen response element-mediated transcription, and intracellular increases in native estradiol and estrone-3-sulfate, confirmed both by ELISA and MALDI-IMS, relative to the GLP-1-CEX backbone. Further, we identify VATPase-mediated endolysosomal acidification as crucial for estrogenic activity following GLP-1-CEX/E2 internalization, suggesting that lysosomal pH-dependent proteolytic enzymes are likely responsible for E2 moiety liberation. However, the extent to which modulating lysosomal acidification – or other optimization strategies – can replicate the estrogenic activity of freely diffusible E2 remains to be seen, but presents an exciting opportunity given the sustained localization of E2 in the intracellular space due to GLP-1R-mediated anchoring. The amide/ether site on the GLP-1-CEX/E2 linker likely favors the formation of E2-3-ether following proteolytic cleavage, rather than native estradiol. Although we did not detect free intracellular E2-3-ether, we demonstrate its preferential conversion into estrone-3-sulfate. Our findings show that GLP-1-CEX/E2 co-administration with VATPase activator EN6 increases intracellular estradiol accumulation, and to a greater degree, estrone-3-sulfate accumulation. Based on previous literature illustrating substantial conversion of estrone-3-sulfate to estradiol [[31], [32], [33], [34]], we suggest that the increase in E2 from GLP-1-CEX/E2 administration likely derives from estrone-3-sulfate accumulation originating from E2-3-ether metabolism. These insights may enhance our understanding of E2 liberation dynamics at the amide/ether linker within intracellular GLP-1R signaling and endosomal trafficking.

A limitation of this study is the inability to immediately detect E2-3-ether following lysosomal hydrolytic cleavage within the amide/ether linker. The absence of this metabolite might be due to its rapid conversion into canonical metabolites within the estrogen metabolism pathway, and is beyond the resolution of our current approach. Additionally, estrogenic activity of GLP-1-CEX/E2 might be overrepresented in transient GLP-1R overexpression systems relative to *in vivo* tissues with endogenous expression. Nonetheless, mice with CNS-specific knockout of GLP-1R or whole-body knockout of ERα lack GLP-1-CEX/E2-induced synergistic effect on body weight reduction [7], suggesting that endogenous GLP-1R expression within *in vivo* dosing strategies may be sufficient for mediating the estrogenic workflow highlighted in this paper. Finally, it is possible that multiple enzymes are involved in both linker cleavage and subsequent metabolite processing, potentially leading to different metabolic pathways that result in the accumulation of E2, estrone, and estrone-3-sulfate.

Our current findings aim to improve the understanding of small molecule targeting and GLP-1/small molecule conjugate efficacy. While the future of anti-obesogenic strategies using small-molecule conjugation to GLP-1 is promising, further studies are needed to better understand how modifications to the peptide carrier, linker type, selected small molecule, and targeted cell type may influence effective intracellular cargo delivery via GPCR endocytosis. Such studies could enable rationale engineering of next-generation conjugates with more optimal drug-like properties, and support more precise understanding of the pharmacokinetic/pharmacodynamic relationship and translational aspects of these investigational drugs.

## 4 Methods

### 4.1 Plasmids

Untagged human GLP-1R was purchased from Sino Biological Inc. (Cat #: HG13944-UT, Beijing, China). SNAP-GLP-1R was purchased from CisBio, now Revvity Inc. Human GLP-1R-GFP was a gift from Professor David Hodson (University of Birmingham, Birmingham, UK). Human GLP-1R-Rluc8 (hGLP-1R-Rluc8) was a gift from Professor Patrick Sexton (Monash University, Melbourne, Australia). cAMP sensor pcDNA3L-His-CAMYEL (ATCC MBA-277TM) was purchased from ATCC (Manassas, VA, USA) [35]. NES-Nluc-miniG plasmids (mGα_s_ and mGα_q_) were gifts from Kevin Pfleger (Harry Perkins Institute of Medical Research, Nedlands, WA, Australia) as originally published by Professor Nevin Lambert (Augusta University, Augusta, GA, USA) [36]. β-arrestin 1/2-Rluc8 plasmids were a gift from Professor Terry Hébert (McGill University, Montreal, Canada). EGFP-CAAX was a gift from Lei Lu (Addgene plasmid # 86056). Lamp1-mNeonGreen was a gift from Dorus Gadella (Addgene plasmid # 98882). pEF1a-2xSV40_NLS-NLuc was a gift from Antonio Amelio (Addgene plasmid # 135953). ER-mNeonGreen was a gift from Dorus Gadella (Addgene plasmid # 137804). mEmerald-LaminB1-10, mEmerald-Rab4a, mEmerald-Rab7a and mEmerald-Rab11a were gifts from Michael Davidsons (Addgene plasmid # 54140, # 54242, # 54244, # 54245). pCMV-hERα was a gift from Elizabeth Wilson (Addgene plasmid # 101141) as originally published by Professor David J Shapiro [37]. pcDNA Flag ERβ was a gift from Harish Srinivas (Addgene plasmid # 35562). 3X ERE TATA luc was a gift from Donald McDonnell (Addgene plasmid # 11354). pcDNA3.1(+)-ExRai-AKAR2 was a gift from Jin Zhang (Addgene plasmid # 161753). pCMV-GFP was a gift from Connie Cepko (Addgene plasmid # 11153). Plasmids eGFP-Rab35, eGFP-Rab22, eGFP-Rab10, eGFP-Rab1a, eGFP-Rab3a were gifts from Marci Scidmore (Addgene plasmid # 49552, # 49600, # 49472, # 49467, # 49542).

### 4.2 Peptide synthesis

GLP-1-CEX, GLP-1-CEX/E2 and E2-3-ether were provided by Novo Nordisk (Indianapolis, Indiana, USA), and synthesized as previously published [7]. hGLP-1 (7-36) amide was purchased from Anaspec (Cat #: AS22463, Fremont, CA, USA).

### 4.3 Cell culture

HEK293T cells and MIN6 cells were cultured in T175 cell culture flasks (Sarstedt, Cat. No 83.3912.302). HEK293T cells were cultured in DMEM (Life Technologies, Cat. No 31966047) containing 10% heat-inactivated-foetal bovine serum (HI-FBS) (Gibco, Cat. No 10500064) and 1% Penicillin/Streptomycin (Pen/Strep) (Cat. No 5000956). MIN6 cells in DMEM containing 15% HI-FBS, 1% Pen/Strep, 1% l-Glutamine (Gibco, Cat. No 25030-024), 2% HEPES (Gibco, Cat. No 15630-056), and 50 β-mercaptoethanol (Sigma Aldrich, Cat. No 60-24-2). Cells were kept in an incubator at 37 °C with 5% CO2.

### 4.4 Ligand-induced bioluminescence resonance energy transfer (BRET) assays

Cells were seeded at 700K cells per well in 6-well plates (Costar, Cat. No 3335). After 24 h plasmids were overexpressed using Lipofectamine 2000 (Invitrogen, Cat. No 11668-019) as per the manufacturer's instructions. The next day cells were washed with phosphate buffered saline (PBS) (Gibco, Cat. No 10010-015) and resuspended in Fluorobrite (Gibco, Cat. No A1896702) supplemented with 5% HI-FBS and 1% l-glutamine, before being seeded at 100k cells per well into a poly-d-lysine (Sigma Aldrich, Cat. No P6403-10 MG) coated 96-well plate (Thermofisher scientific, Cat. No 136101). The following day baseline fluorescence and luminescence readings were recorded with a PHERAstar FSX plate reader, using Renilla luciferase (Rluc) substrate coelenterazine (Biomol, Cat. No ABD-21165) or Nanoluc (Nluc) substrate furimazine (Promega, Cat. No N113A). The luciferase substrate was added to Hanks Balanced Salt Solution (Sigma, Cat. No 56648) before being added to cells. After recording baseline, agonists were applied and reading completed for a set number of cycles at an adjusted cycle time. A gain adjustment was performed to obtain the correct gain value for each experiment. Experiments were performed three times on three separate biological replicates. All ligand responses over time were normalized to the vehicle group and first two cycle readings. Dose–response negative or positive iAUC data (depending on assay type) is expressed as a log normalized means ± SEM, followed by three-parameter non-linear regression. Statistical analyses were carried out using GraphPad Prism (GraphPad, San Diego, CA, USA) for all experiments, including one-way ANOVA corrected with Tukey's multiple comparison correction.

### 4.5 Estrogen response element (ERE) luciferase reporter assays

Cells were cultured, seeded in 6-well plates, transfected and re-seeded in 96-well plates as for BRET assays. pCMV-hERα as well as the 3X ERE TATA luc was overexpressed in HEK293T cells to allow for E2 induced transcriptional effects. Concomitant CMV-GFP overexpression allowed for normalization of cell count within each well. On the fourth day, cells were provided Fluorobrite without FBS for 1 h before agonists were administered, and the plates incubated for 24 h. The next day GFP fluorescence was recorded using the PHERAstar FSX plate reader and Bright-glo reagent (Promega, Cat. No E2620). Luciferase values were divided by GFP values to obtain normalised results. Experiments were performed at least three times on three separate biological replicates, different wells were considered technical replicates.

### 4.6 PKA activity assay

Cells were cultured, seeded in 6-well plates and transfected as for the BRET assay but re-seeding was done into black 96-well plates (Fisher Scientific Cat. No 655077). In this case the hGLP-1R-untagged and ExRai-AKAR2 were overexpressed in these cells. Gain adjustment was performed for each experiment. Fluorescence intensity ratio was calculated instead of ligand-induced BRET ratio. Baseline was subtracted as for the BRET measurements. Data analysis was performed as for the BRET measurements.

### 4.7 Immunocytochemistry (ICC)

Cells were seeded in a poly-d-lysine coated 8-well slide (Lab-Tek, Cat. No 177445) at 80K cells per well. The following day cells were transfected with Lipofectamine2000 according to the manufacturer's instructions. SNAP-GLP-1R was overexpressed in these cells. Cells were kept in Fluorobrite and cultured in an incubator held at 37 °C and at 5% CO2. After 24 h cells were incubated with SNAP-Surface Alexa Fluor 488 (New England Biolabs, Cat. No S9129S) for 30 min at 5 μM. Thereafter, cells were washed with PBS and treated with agonists in Fluorobrite reagent. In a time-dependent fashion, cells were fixed with 4% paraformaldehyde (PFA) solution (ThermoScientific, Cat. No J19943) every 20 min. Wells were then washed with PBS and kept in PBS at 4 °C. Permeabilization was carried out for 10 min after cells were washed with TBS. Blocking was done with 5% Donkey Serum, and cells were washed with TBS. Primary staining was then performed using an E2 antibody (Revmab, Cat. No 31-1230-00) at 1:200 and cells were left incubating with the primary antibodies overnight at 4 °C. The next day secondary staining was done with the appropriate antibody (Donkey-anti rabbit Alexa 594) at 1:1000 before nuclear staining was performed with DAPI. The slides were mounted with a coverslip using DAKO mounting medium (Agilent, Cat. No S3023). Slides were imaged at ∗60 Magnification using a SP-8 confocal microscope. Z-stacks were obtained for each field of view (FOV). Analysis was carried out using FIJI/ImageJ. During the analysis each cell was isolated and duplicated, then the channels separated, and the coloc2 tool was used to correlate the E2-ab channel to the SNAP-GLP-1R channel. Mander's coefficient was obtained for each cell expressing DAPI and SNAP-GLP-1R markers. Experiments were performed at minimum three times.

### 4.8 Proximity ligation assay (PLA)

Cells were cultured, seeded, transfected, treated, and fixed as per for the IHC experiment. SNAP-GLP-1R and pCMV-hERα were overexpressed in HEK293T cells during these experiments. Primary staining was performed with SP-1 (Abcam, Cat. No ab124804) and ERα (Abcam, Cat. No 259427) antibodies at 1:200, and the 8-well slide incubated at 4 °C overnight. During the secondary staining Duolink PLA (Sigma Aldrich, Cat. No DUO92007) protocol was followed as per manufacturer's instructions. DAPI staining and mounting was performed as in the IHC protocol. Imaging was carried out at ∗60 Magnification using a SP-8 confocal microscope. Z-stacks were obtained for each ROI and analysed with FIJI/ImageJ. The Z-Stack for each cell was duplicated, the channels separated, and the signal intensity for the PLA channel was measured across five images for each nucleus, an average signal intensity for the five images was taken for each nucleus. Experiments were performed three times.

### 4.9 17β-estradiol ELISA and estrone-3-sulfate ELISA

Cells were seeded in 6-well plates where the hGLP-1R-untagged was overexpressed using Lipofectamine2000, following manufacturer's instructions. Then, wells were treated with different agonists for a 2-h period. The cells were washed twice with PBS and then lysed with RIPA buffer (Sigma, Cat. No R0278) containing proteinase and phosphatase inhibitors (Thermo Scientific, Cat. No 78446) to obtain the cellular lysate. The lysate was sonicated for 10 min and centrifuged at 17,000g for 20 min to remove the cell debris. Both ELISAs (Abcam, 108667, Thermofisher Scientific, EIA17E3S) were performed using this cell lysate. Experiments were performed on six biological replicates.

### 4.10 Matrix assisted laser desorption ionisation-imaging mass spectrometry (MALDI-IMS)

The MALDI-FTICR MSI measurement was performed as previously described [38]. Cryosections of 12 μm thickness from cell pellets were prepared using a cryotome (CM1950, Leica Microsystems, Wetzlar, Germany). These tissue sections were thaw-mounted on indium-tin-oxide-coated conductive glass slides (Bruker Daltonik, Bremen, Germany). The tissue sections were spray-coated with 10 mg/mL of 9-aminoacridine hydrochloride monohydrate matrix (Sigma–Aldrich) in 70% methanol using the SunCollect sprayer (Sunchrom, Friedrichsdorf, Germany). The matrix was applied in 8 passes, with ascending flow rates of 10 μL/min, 20 μL/min, and 30 μL/min for layers 1–3, and 40 μL/min for layers 4–8. The MALDI-MSI measurements were conducted in negative-ion mode on a 7T Solarix XR Fourier-transform ion cyclotron resonance mass spectrometer (Bruker Daltonik, Bremen, Germany) equipped with a dual electrospray ionization–MALDI (ESI-MALDI) source and a SmartBeam-II Nd:YAG (355 nm) laser. Mass spectra were acquired over a mass range of *m*/*z* 75–1100. The instrument was calibrated externally with l-arginine in the ESI mode and internally using the 9-AA matrix ion signal (*m*/*z* 193.0771) as a lock mass. The laser operated at a frequency of 1000 Hz using 100 laser shots per pixel with a spatial resolution of 70 μm. After MALDI-MSI, the cell pellet sections were stained with hematoxylin-eosin, and scanned with an AxioScan.Z1 digital slide scanner (Zeiss, Jena, Germany) equipped with a 20 × magnification objective. The acquired MS data were subjected to spectral processing in FlexImaging v. 5.0 (Bruker Daltonics, Bremen, Germany) and SCiLS Lab v. 2024 (Bruker Daltonics, Bremen, Germany). Mass spectrometry peaks were annotated using the Human Metabolome Database (HMDB, https://www.hmdb.ca/) [39], METASPACE (https://metaspace2020.eu) [40], and the KEGG database (https://www.genome.jp/kegg/) [41].

## CRediT authorship contribution statement

**Callum Coupland:** Writing – original draft, Investigation, Formal analysis, Data curation. **Na Sun:** Formal analysis, Data curation. **Ahmed Khalil:** Formal analysis, Data curation. **Özüm Ezgi Karaoglu:** Formal analysis, Data curation. **Arkadiusz Liskiewicz:** Supervision, Investigation, Formal analysis. **Daniela Liskiewicz:** Supervision, Investigation, Formal analysis. **Gerald Grandl:** Supervision, Formal analysis. **Seun Akindehin:** Investigation, Formal analysis. **Gandhari Maity:** Investigation, Formal analysis. **Bin Yang:** Formal analysis, Data curation. **Brian Finan:** Writing – review & editing, Project administration, Methodology, Formal analysis. **Patrick Knerr:** Writing – review & editing, Project administration, Formal analysis. **Jonathan D. Douros:** Writing – review & editing, Supervision, Resources, Methodology, Investigation, Formal analysis, Data curation. **Axel Walch:** Formal analysis, Data curation. **Richard DiMarchi:** Writing – review & editing, Supervision. **Matthias H. Tschöp:** Writing – review & editing, Supervision. **Timo D. Müller:** Writing – review & editing, Writing – original draft, Validation, Supervision, Resources, Investigation, Funding acquisition, Formal analysis, Conceptualization. **Aaron Novikoff:** Writing – review & editing, Writing – original draft, Supervision, Methodology, Investigation, Formal analysis, Data curation, Conceptualization.

## Declaration of competing interest

The authors declare the following financial interests/personal relationships which may be considered as potential competing interests:

MHT is a member of the scientific advisory board of ERX Pharmaceuticals, Cambridge, Mass. He was a member of the Research Cluster Advisory Panel (ReCAP) of the Novo Nordisk Foundation between 2017 and 2019. He attended a scientific advisory board meeting of the Novo Nordisk Foundation Center for Basic Metabolic Research, University of Copenhagen, in 2016. He received funding for his research projects by Novo Nordisk (2016-2020) and Sanofi-Aventis (2012-2019). He was a consultant for Bionorica SE (2013-2017), Menarini Ricerche S.p.A. (2016), and Bayer Pharma AG Berlin (2016). As former Director of the Helmholtz Diabetes Center and the Institute for Diabetes and Obesity at Helmholtz Zentrum München (2011-2018), and since 2018, as CEO of Helmholtz Zentrum München, he has been responsible for collaborations with a multitude of companies and institutions, worldwide. In this capacity, he discussed potential projects with and has signed/signs contracts for his institute(s) and for the staff for research funding and/or collaborations with industry and academia, worldwide, including but not limited to pharmaceutical corporations like Boehringer Ingelheim, Eli Lilly, Novo Nordisk, Medigene, Arbormed, BioSyngen, and others. In this role, he was/is further responsible for commercial technology transfer activities of his institute(s), including diabetes related patent portfolios of Helmholtz Zentrum München as, e.g., WO/2016/188932 A2 or WO/2017/194499 A1. MHT confirms that to the best of his knowledge none of the above funding sources were involved in the preparation of this paper. TDM receives research funding by Novo Nordisk but these funds are unrelated the here described work. R.D.D is a co-inventor on intellectual property owned by Indiana University and licensed to Novo Nordisk. He was previously employed by Novo Nordisk. B.F. is currently an employee of Eli Lilly. TDM receives funding from Novo Nordisk and received speaking fees within the last 4 years from Novo Nordisk, Eli Lilly, AstraZeneca, Merck, BerlinChemie AG, and Mercodia. B.Y., B.F., P.J.K., and J.D.D. are former employees and shareholders of Novo Nordisk. If there are other authors, they declare that they have no known competing financial interests or personal relationships that could have appeared to influence the work reported in this paper.

## Data Availability

Data will be made available on request.
